# FGF2 modulates simultaneously the mode, the rate of division and the growth fraction in cultures of radial glia

**DOI:** 10.1242/dev.189712

**Published:** 2020-07-24

**Authors:** Mario Ledesma-Terrón, Nuria Peralta-Cañadas, David G. Míguez

**Affiliations:** Departamento de Física de la Materia Condensada, Instituto de Física de la Materia Condensada, IFIMAC, Instituto Nicolas Cabrera, INC, Centro de Biología Molecular Severo Ochoa, CBMSO, Universidad Autónoma de Madrid, Madrid 28012, Spain

**Keywords:** Cell cycle, Differentiation, Branching processes, Radial glia, Thymidine analogs

## Abstract

Radial glial progenitors in the mammalian developing neocortex have been shown to follow a deterministic differentiation program restricted to an asymmetric-only mode of division. This feature seems incompatible with their well-known ability to increase in number when cultured *in vitro*, driven by fibroblast growth factor 2 and other mitogenic signals. The changes in their differentiation dynamics that allow this transition from *in vivo* asymmetric-only division mode to an *in vitro* self-renewing culture have not been fully characterized. Here, we combine experiments of radial glia cultures with numerical models and a branching process theoretical formalism to show that fibroblast growth factor 2 has a triple effect by simultaneously increasing the growth fraction, promoting symmetric divisions and shortening the length of the cell cycle. These combined effects partner to establish and sustain a pool of rapidly proliferating radial glial progenitors *in vitro*. We also show that, in conditions of variable proliferation dynamics, the branching process tool outperforms other commonly used methods based on thymidine analogs, such as BrdU and EdU, in terms of accuracy and reliability.

## Abstract (precis)

**Highlighted Article:** When mode and/or rate of division are changing, a branching process, rather than a thymidine analog method, provides temporal resolution, it is more robust and does not interfere with cell homeostasis.

## INTRODUCTION

The neocortex constitutes the main part of the mammalian brain, and the location where the processing of all higher-order brain functions resides. Understanding its formation is one of the major interests in the field of developmental biology (Lodato and Arlotta, 2015). The neocortex develops from a stratified neuroepithelium, called the neural tube, into a complex structure of six horizontal layers of excitatory and inhibitory neurons (Matsuzaki and Shitamukai, 2015). Neurogenesis in the developing neocortex initiates when self-renewing neuroepithelial progenitors (NEPs) transform into apical and basal radial glia (RG) progenitor cells and start to produce neurons and intermediate neuronal precursors (Beattie and Hippenmeyer, 2017; Taverna et al., 2014). Since the discovery that RG constitute the progenitors of potentially all neurons in the vertebrate neocortex (Frederiksen and McKay, 1988; Hartfuss et al., 2001; Miyata et al., 2001; Noctor et al., 2004), a great effort has been focused in identifying their features and properties: how they coordinate in time and space to form the multiple layers of the neocortex; which signals control their fate; and how these signals orchestrate the correct balance between proliferation or differentiation during neurogenesis.

In principle, this balance can be robustly achieved via stochastic or deterministic cell decisions (Losick and Desplan, 2008). In brief, stochastic models assume a certain probability of differentiation that depends on the intracellular and extracellular signals that the cell is receiving. In this context, the fate at the single cell level is unpredictable and the balance between proliferation and differentiation is regulated at the level of the population (Teles et al., 2013). On the other hand, deterministic models of stem cell differentiation assume that the fate of the progeny is fixed and, therefore, the correct balance between the numbers of different types of neurons is achieved at the single cell level (Müller-Sieburg et al., 2002).

The dynamics of differentiation is often characterized based on the fate of the two daughter cells of a cell division relative to each other (Kosodo et al., 2004). This way, proliferating progenitors can perform *pp* (progenitor-progenitor), *pd* (progenitor-differentiated) and *dd* (differentiated-differentiated) divisions (Huttner and Kosodo, 2005). In this context, differentiation in the developing chick spinal cord (Míguez, 2015), in the zebrafish retina (He et al., 2012; Chen et al., 2012), epidermis (Clayton et al., 2007), airway epithelium (Teixeira et al., 2013), germline (Klein et al., 2010) and the intestine (Snippert et al., 2010) of mice follow a stochastic model. In these systems, progenitors can potentially perform each of the three types of division, and the corresponding rates are probabilistic and change overtime. On the other hand, the differentiation of RG in the mammalian brain has been shown to follow a deterministic asymmetric-only mode of division (Gao et al., 2014; Beattie and Hippenmeyer, 2017).

Several years ago, the group of Austin Smith showed that RG extracted from mouse developing neocortex can be successfully cultured *in vitro* (Conti et al., 2005). Driven by the multiple phenotypic similarities between neuronal precursors differentiated from embryonic stem cells in culture and RG, authors suggested that these neuronal precursors are the culture analogs to RG. In the same paper and driven by this observation, they also showed that *in vitro* cultures of RG could be established with fibroblast growth factor 2 (FGF2) and EGF as the key molecules that facilitate their expansion (Conti et al., 2005).

FGF2 is an extensively studied neurogenic factor for proliferation and differentiation of multipotent neural stem cells both during development and in the adult mouse brain (Kang and Hébert, 2015). FGF2 has been shown to be necessary for cell proliferation and neurogenesis *in vivo*, and to induce additional mitoses in progenitor cells *in vitro* (Raballo et al., 2000). In addition, stem cells from the adult mouse brain have been shown to proliferate and self-renew *in vitro* in the presence of FGF2 (Gritti et al., 1996). On the other hand, FGF2 stimulation have been shown to control the fate, migration and differentiation but not the proliferation of neuronal progenitors *in vivo* (Dono et al., 1998), whereas more recent studies do show an impact in promoting the cell cycle progression in cultures of rat glioblastoma cells (Baguma-Nibasheka et al., 2012).

From all these potential effects of FGF2, the specific features that facilitate the transition of RG from a non-expanding population *in vivo* that can perform only asymmetric *pd* divisions (and is, therefore, incompatible with progenitor cell expansion in number), to a self-renewing *in vitro* culture have not been quantitatively characterized in detail. In principle, this transition can be achieved by reducing the rate of neurogenesis, by promoting proliferative (at the expenses of asymmetric or symmetric differentiative) divisions, by increasing the proliferation rate (by shortening the cell cycle), by inducing cell cycle re-entry of quiescent progenitors (i.e. increasing the growth fraction), by reducing apoptosis (as a pro-survival signal), by inducing intermediate progenitors (that perform additional terminal divisions) or by shifting RG towards its less mature NEP phenotype (that perform *pp* divisions *in vivo*).

In this paper, we quantify the specific effects of FGF2 on key features of the proliferation and differentiation dynamics of RG that allow them to be cultured and expanded *in vitro*. To achieve this, we quantify values of cell numbers of RG and differentiated neurons extracted from mouse developing cerebral cortex and cultured in the presence of different FGF2 concentrations and at different time points. These values inform a theoretical framework based on a branching process formalism (Míguez, 2015) that provides average values of mode and rate of division of the RG population with temporal resolution. Our results show that FGF2 does not affect the rate of neurogenesis (i.e. the amount of differentiated neurons produced), it does not promote the NEP or intermediate progenitor phenotype and it does not affect significantly the apoptosis rate. On the other hand, FGF2 does promote symmetric *pp* divisions, it increases the growth fraction and shortens the average cell cycle length. These three key effects when combined, strongly facilitate the propagation and expansion of the culture.

In addition, discrepancies between predictions for the cell cycle length and growth fraction using several methods in our study pointed us to compare the accuracy of several common methodologies used to measure cell cycle features. To do that, we use a numerical model to show that methods based on cumulative thymidine analogs (such as EdU and BrdU) are not accurate in conditions of variable differentiation dynamics. On the other hand, the method based on branching process formalism performs better when mode and/or rate of division are changing, which is the case in our RG cultures and many in other *in vivo* developmental systems. In addition, the branching process method is superior due to its temporal resolution, robustness, minimal interference with cell homeostasis and simplicity of use.

## RESULTS

### FGF2 stimulation increases the growth rate of cultures of RG by shortening the length of the cell cycle

**Fig. 1.**
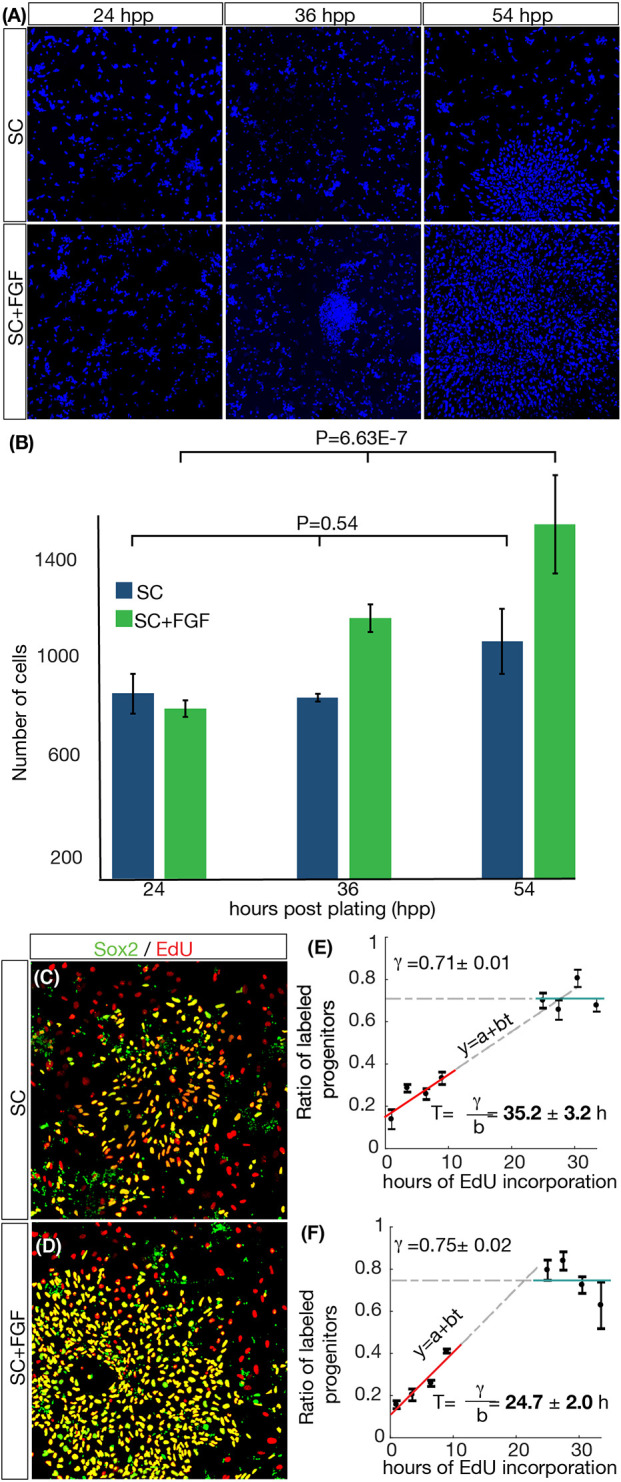
**FGF2 shortens the division time of cycling RG *in vitro*.** (A) Snapshots of RG cultures at different hours post-plating (hpp) stained with Hoechst and growing under SC and SC+FGF culture conditions. (B) Total cell numbers in a field of view of 0.6 mm×0.6 mm at different time points. Error bars correspond to s.e.m. between multiple samples of similar conditions. (C,D) Sox2 (green) and EdU (red) staining to mark progenitors that have gone through S phase in 24 h of EdU incorporation. (E,F) Cumulative curve of EdU-positive progenitors shows that cells in SC+FGF conditions cycle faster (*T*=24.7±2.0 h) than in SC (*T*=35.2±3.5 h), while the growth fraction *γ* remains similar.

To initially test how the dynamics of growth and differentiation of RG *in vitro* is modulated by FGF2, cells derived from the developing neocortex of mouse embryos at E11-E11.5 were extracted, plated and cultured following standard protocols (Hilgenberg and Smith, 2007). Starting at 24 h post-plating (hpp), samples were fixed at three different time points and stained with Hoechst (Fig. 1A). Quantification of the number of cells in a field of view of fixed dimensions (0.6 mm×0.6 mm) using an automated segmentation tool developed in house (see Materials and Methods) is shown in Fig. 1B for two culture conditions, SC and SC+FGF, where the standard culture media is supplemented with an increased concentration of FGF2 ligand (see Materials and Methods). In both conditions, the number of cells increases, but the growth is only statistically significant (*P*<0.05) in SC+FGF conditions.

To study in detail how FGF2 affects the length of the cell cycle of the cycling progenitors, we performed 5-ethynyl-2′-deoxyuridine (EdU) cumulative labeling experiments to measure changes in the length of the average cell cycle. BrdU (Nowakowski et al., 1989), EdU (Salic and Mitchison, 2008; Buck et al., 2008) and other thymidine analogs constitute the most-used tools for estimating the cell cycle length of cells in many contexts (Alexiades and Cepko, 1996). The method is based on the replacement of endogenous thymidine during DNA synthesis with traceable compounds (Takahashi, 1966; Takahashi et al., 1996). The length of the average cell cycle is then inferred from the dynamics of the incorporation of these compounds into the DNA of cycling cells (Macdonald, 1970).

To estimate the average cell cycle length of the population, samples were cultured in the presence of EdU and then fixed at different time points (corresponding to different times of EdU incorporation). Combined nuclear Hoechst staining with EdU detection assay and immunostaining for Sox2 were used to identify all progenitors that had passed through S phase for each EdU incubation time.

The cell cycle length *T* and the growth fraction *γ* were calculated using the standard cumulative curve method based on linear regression (see Materials and Methods). Representative snapshots are shown in Fig. 1C-F. The resulting cumulative curves (Fig. 1E,F) reveal that *γ* remains at around 72% for both conditions tested, while *T* depends strongly on the culture conditions (*T*=35.2±3.2 h for SC, *T*=24.7±2.0 h for SC+FGF). In conclusion, our results show that FGF2 stimulation shortens the average cell cycle length in cultures of RG *in vitro*, while its effect in the growth fraction is not statistically significant.

### FGF2 stimulates the generation of progenitors in culture

The previous section shows that FGF2 affects the rate of division. To study the effect of FGF2 in the number of cells of each specific population of RG progenitors and differentiated neurons, we extracted the neocortex of mouse embryos at E11-E11.5 and plated cells at the same initial cell density in different wells. Next, cells were cultured under the two FGF2 conditions and samples were fixed every 2-4 h, starting at 24 h post-plating (hpp). Next, samples were stained using antibodies against Sox2 and Map2 to identify progenitors and differentiated cells, respectively. We then identified the fate of each cell based on the intensity of Sox2 and Map2 staining using our segmentation framework (see Materials and Methods).

**Fig. 2.**
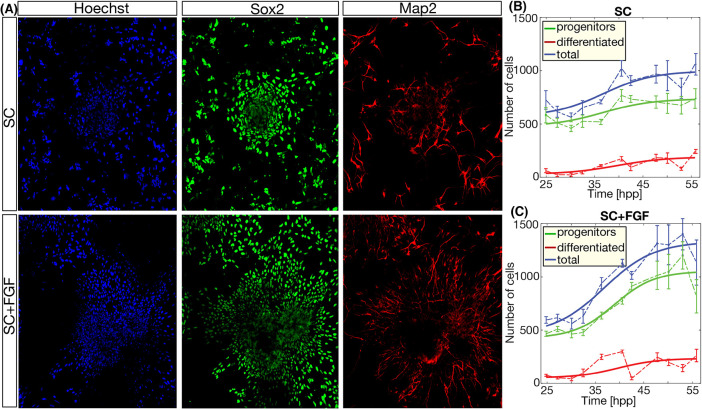
**FGF stimulation increases the amount of progenitor cells.** (A) Snapshots of RG cultures at 24 h post-plating showing nuclei (Hoechst), progenitors (stained for Sox2) and differentiated neurons (stained for Map2). (B,C) Quantification of the number of cells of each type in both culture conditions at different time points shows an increased number of progenitors in SC+FGF, compared with SC conditions. Error bars correspond to s.e.m. Lines correspond to nonlinear sigmoidal fitting of the experimental data points.

Results are shown in Fig. 2A. Output provided by the segmentation script is plotted in Fig. 2B,C. Assuming the typical logistic growth model (Juarez et al., 2016) for proliferating cells in cultures, the corresponding sigmoidal curve fitting is also plotted (green, red and blue lines for RG, neurons and total cells, respectively). The data show that an initial regime of reduced change in cell numbers is followed by an increase in both cell types until the system reaches a regime where few new cells are being generated. Under both conditions, the amount of progenitors (green data points, green line) and differentiated cells (red data points, red line) increases with statistical significance (*P*<0.05) but the increase in progenitors is statistically more significant in conditions of SC+FGF (*P*=7.25E-09) that in SC conditions (*P*=7.60E-03).

In principle, this increase in the progenitor population could be explained by an increase in neuroepithelial progenitors (NEPs) (Beattie and Hippenmeyer, 2017; Taverna et al., 2014) or intermediate progenitors (Molyneaux et al., 2007), which emerge from asymmetric division of the RG and are able to perform a terminal *dd* division (Hutton and Pevny, 2011). Immunofluorescence against Tbr2, a marker for intermediate progenitors, shows no Tbr2-positive cells in the two culture conditions tested (Fig. S1C). This is in agreement with the effect of FGF2 in inhibiting the transition from RG to intermediate progenitor (Kang et al., 2009) (FGF2 is in the culture media in both experimental conditions: SC and SC+FGF). Quantification of immunofluorescence against Pax6, a well-characterized marker for RG (Suter et al., 2009) that is not present in NEPs (Elsen et al., 2018), shows that close to 100% of all Sox2-positive progenitors are also positive for Pax6 (Fig. S1A), suggesting that FGF2 stimulation does not promote the transition of RG back to neuroepithelial progenitors (Englund et al., 2005).

In conclusion, the increase in FGF2 concentration results in more RG and similar number of differentiated cells, showing that the population of cycling progenitors does not grow at the expense of the terminally differentiated cells.

### Branching process formalism predicts variable mode of division that is affected by FGF2 stimulation

The previous observation suggests that, apart from the changes in the cell cycle length, FGF2 may also be affecting the mode of division of the RG. It has been shown previously that the fate of differentiating RG can be modulated by FGF2, by changing the differentiation progeny of RG from neurons to glia (Qian et al., 1997). To quantify the effect of FGF2 in the mode of division, we took advantage of a branching process theoretical formalism developed by our lab (Míguez, 2015). In brief, the tool provides the average rates of each mode of division with temporal resolution simply based on numbers of progenitors and differentiated cells at different time points (see Materials and Methods).

Input data of the framework are the numbers of progenitors and differentiated cells, the rate of apoptosis and the growth fraction. To obtain the average rate of apoptosis, we performed immunostaining against anti-cleaved caspase 3 at three time points in the cultures under SC and SC+FGF conditions. Comparison between both conditions showed a very reduced rate of apoptosis that is not significantly affected by the addition of extra FGF2 (Fig. S1B).

**Fig. 3.**
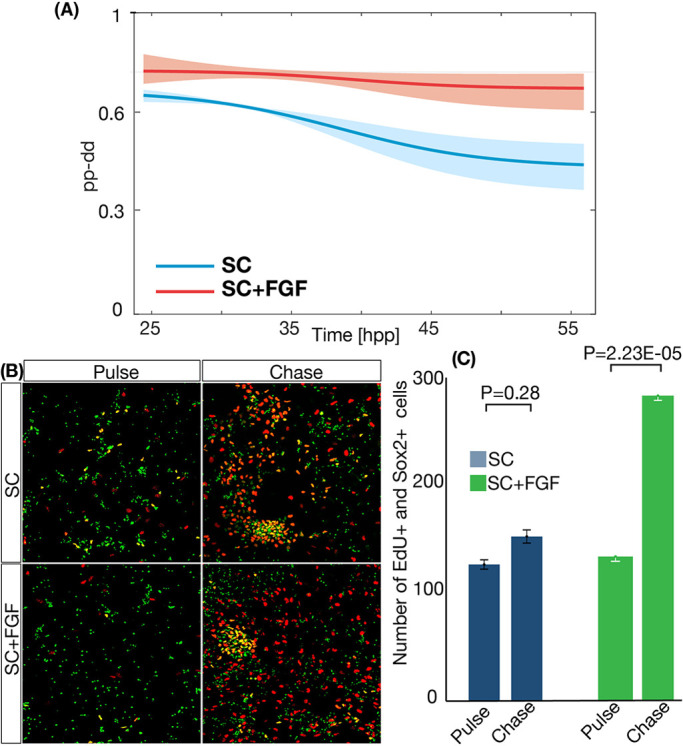
**FGF affects the proportion of symmetric proliferative divisions in RG culture.** (A) Plot of the average value of *pp*−*dd* of the population of RG under SC (red) and SC+FGF (blue) conditions. Areas around the curves represent the 50% confidence interval. (B) Representative images showing Sox2 and EdU (stained in green and red, respectively) for ‘pulse’ and ‘chase’ time points. (C) Quantification of the number of Sox2- and EdU-positive cells for time-points for SC and SC+FGF conditions. Error bars correspond to s.e.m. between independent repeats of the experiment.

Next, the apoptosis rate and the fitted values of cell numbers for progenitors and differentiated cells were used to estimate the average mode of division. This is provided by Eqn 1 in the Materials and Methods (Míguez, 2015) in terms of the difference between the rates of *pp* and *dd* divisions (*pp−dd*). Results are shown in Fig. 3A. Interestingly, both conditions of SC (blue) and SC+FGF (red) show values of *pp*−*dd*≠0, which would correspond to the *in vivo* situation of asymmetric-only divisions *pd*=1 (as *pp*+*pd*+*dd*=1). In addition, the average rate of differentiation is not constant in time, with the maximum change in the differentiation dynamics occurring around 36-37 hpp, which is more prominent for SC conditions. Comparison between the two curves shows that the value of *pp*−*dd* predicted is higher when more FGF2 is present in the culture media, which corresponds with the higher increase in the number of progenitors observed in SC-FGF conditions (Fig. 2B,C).

To further validate the result that an increase in FGF2 increases the amount of *pp* divisions, we designed an experiment based on pulse and chase of EdU-labeled cells. To do this, we plated cells from mouse developing neocortex following the procedure explained in the Materials and Methods section. Next, cells were cultured under SC and SC+FGF conditions until 33 hpp. At this point, a 30 min pulse of EdU was applied to all samples. A number of samples were fixed at this time point (and labeled as ‘Pulse’ time point). The rest of the samples were washed with fresh culture media five times to remove the EdU (see Materials and Methods). These samples were cultured for another 15 h (corresponding to the predicted average *T* for SC+FGF conditions during this time, to ensure that labeled cells cannot cycle more than once in any of the culture conditions). Next, cells were fixed at this ‘Chase’ time point and stained using Hoechst, EdU and Sox2 immunostaining. Finally, the number of Sox2^+^/EdU^+^ cells at the time of the ‘Pulse’ (33 hpp) and ‘Chase’ (48 hpp) was quantified using our automated image analysis tool (see Materials and Methods). Results are shown in Fig. 3B,C. The number of progenitors labeled with EdU does not change significantly in SC conditions, consistent with a large proportion of asymmetric divisions (i.e. one EdU^+^ RG produces two EdU^+^ cells: one RG and one neuron; so the amount of EdU^+^ RG remains constant) or a balanced ratio between *pp* and *dd*. On the other hand, in conditions of SC+FGF, we see a statistically significant (*P*<0.05) increase in the number of EdU^+^ RG when comparing ‘Pulse’ and ‘Chase’ time points. This result shows that more RG originally labeled with the short EdU pulse divided and produced more RG when FGF2 was increased.

### The length of the cell cycle is variable and shortens in response to FGF2 stimulation

**Fig. 4.**
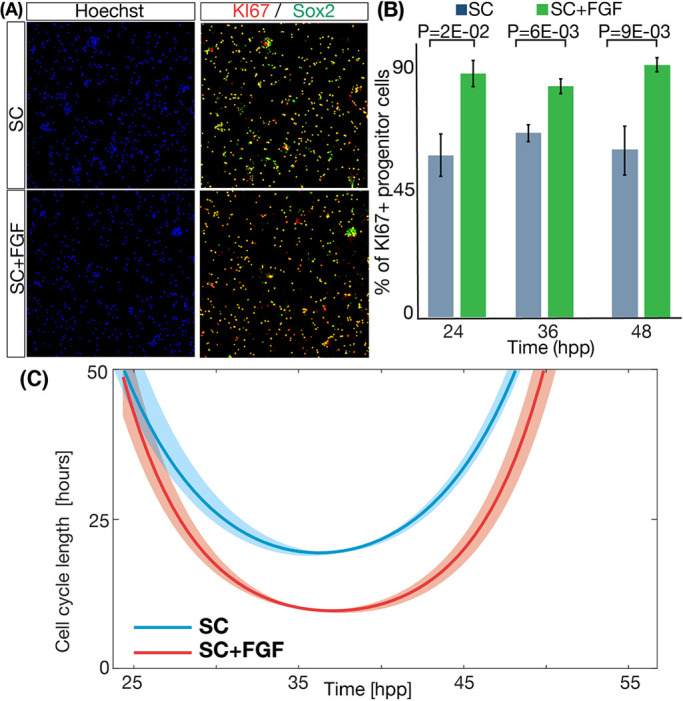
**The growth fraction and the length of the cell cycle change in response to FGF2.** (A) Example of cells stained for nuclei (blue), Ki67 (red) and Sox2 (green) at 36 hpp. (B) Quantification of the percentage of progenitor cells that are actively cycling in both conditions and at three different time points. Columns represent the mean between independent repeats. Error bars represent s.e.m. (C) Cell cycle prediction by the branching process formalism for the two different FGF2 concentrations tested: SC (red) and SC+FGF (blue). Areas around the curves represent the 50% confidence interval.

The branching process formalism also provides the average cell cycle length of the progenitors in the culture with temporal resolution (Eqn 2 in the Materials and Methods). This equation uses as additional input the value of the growth fraction *γ*, which can be indirectly obtained from the EdU experiments in Fig. 1E,F. A more direct estimation of the amount of quiescent progenitors, can be measured by immunofluorescence against KI67 at different time points (Fig. 4A) (Scholzen and Gerdes, 2000). The automated quantification of the number of Sox2^+^ cells that are also KI67^+^ in both culture conditions (Fig. 4B) shows statistically significant differences between SC and SC+FGF conditions, contrary to the results obtained with EdU cumulative curves in Fig. 1E,F. Under SC conditions, the growth fraction is around 55%, while the value in SC+FGF conditions is closer to 90%. This discrepancy between the EdU data (Fig. 1E,F) and the KI67 immunofluorescence (Fig. 4B) is discussed and studied in detail in the next section.

The value of the cell cycle length obtained as output of Eqn 2 is plotted in Fig. 4C, showing an average value of *T* that is not constant: a continuous decrease in cell cycle length is followed by an increase at later time points, and the minimum values for SC (around *T*=19 h) and SC+FGF (around *T*=10 h) conditions occur around 36-37 hpp. This reduction in the cell cycle length is in agreement with previous studies by the Dehay group showing that RG cells accelerate the cell cycle in response to FGF by shortening the length of the G1 phase (Lukaszewicz et al., 2002). In addition, these values are close to the values measured *in vivo* by Gao et al. (2014) and Beattie and Hippenmeyer (2017), who reported an average cell cycle length of 16-18 h in the temporal window corresponding to E11-E13.

In addition, we have observed that some cells in the culture organize in clusters, whereas others remain more isolated. This reorganization suggests that maybe there is heterogeneity in the differentiation dynamics (i.e. cells in clusters can retain apicobasal polarity and therefore behave differently from the rest of the cells in the culture). To test this hypothesis, we compared the change in EdU in the pulse-chase experiment (introduced in Fig. 3B) in cells in clusters versus non-cluster cells. Results are shown in Fig. S5, where the percentage of EdU^+^ cells in both ‘cluster’ and ‘non-cluster’ locations, at both ‘Pulse’ and ‘Chase’ time points, and under both SC and SC+FGF conditions are plotted. Under SC conditions, the increase in EdU^+^ cells in clusters and non-cluster cells is similar, suggesting a similar cell cycle length. Under SC+FGF conditions, the amount of EdU^+^ cells is higher in the ‘Chase’ in cluster cells, but this difference may be due to an already higher number of EdU^+^ cells at the ‘Pulse’ time point already in clusters. In fact, the difference between ‘Pulse’ and ‘Chase’ remains around 40%, suggesting that, also under SC+FGF conditions, the cell cycle is similar between clusters and non-cluster cells.

### The branching process tool outperforms cumulative curve methods to monitor cell cycle dynamics

Interestingly, and despite showing the same trend of shortening *T* with FGF2, the values of the cell cycle length predicted by the branching process formalism do not agree with the ones obtained by the EdU cumulative experiments in Fig. 1E,F. This discrepancy in cell cycle dynamics and in the growth fraction (Fig. 4B) led to us investigate the potential source of conflict between the cumulative method and the branching process tool by developing a numerical model of a generic differentiating stem cell population. A numerical analog of EdU is also simulated computationally, in such a way that cells in S phase are marked as labeled when EdU is present. The number of progenitors, differentiated and EdU-positive progenitors at each time point is used to calculate the average cell cycle length of the population using three widely used EdU-based methods: single cumulative curve (C1) (Nowakowski et al., 1989), dual cumulative (C2) (Shibui et al., 1989) and the pulse-chase (PC) method (Weber et al., 2014). The cell cycle is also calculated using the branching process (BP) method (Míguez, 2015) (Eqn 2 in the Materials and Methods). A detailed description of each method and how it is applied in this context is illustrated in Fig. S3 and explained in the Materials and Methods section. All predictions were then compared with the input value of *T* used for each simulation, to estimate the accuracy and reliability of each method.

**Fig. 5.**
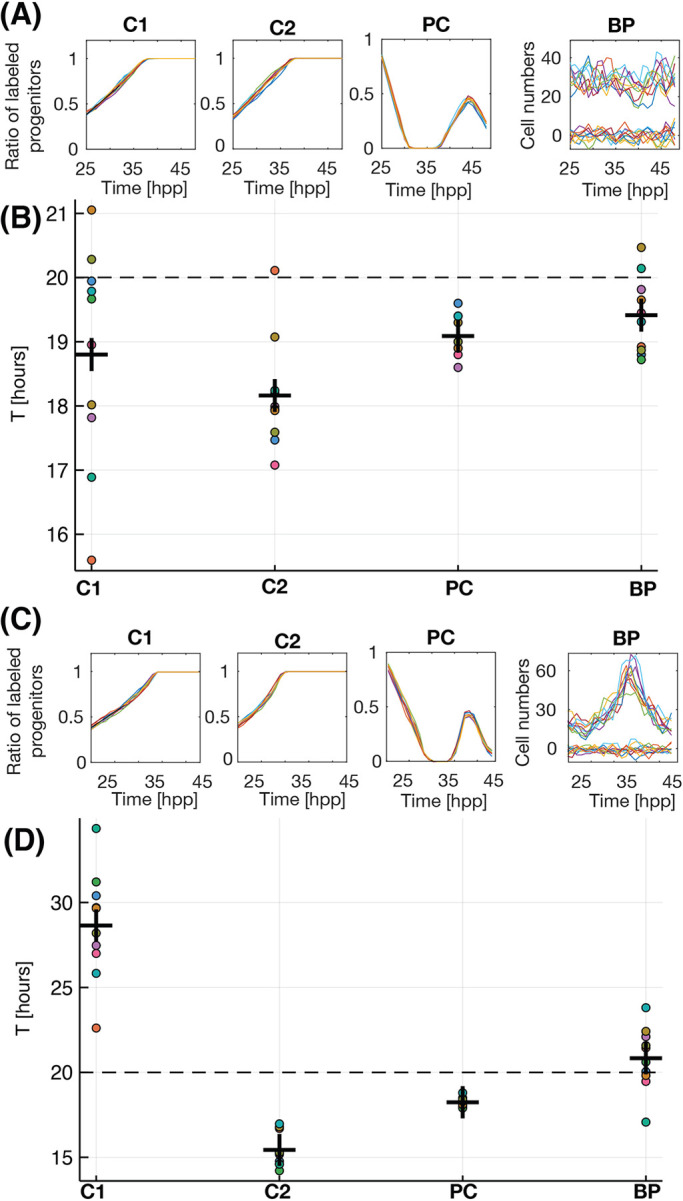
**The branching process tool outperforms cumulative curve methods.** Cumulative curves and quantification for single cumulative (C1), dual cumulative (C2), pulse-chase (PC) and branching process (BP) methods for 10 independent runs of the numerical model for conditions of (A,B) constant and (C,D) variable cell cycle length. Each color corresponds to the same simulation analyzed using each framework (see text). The cell cycle is also calculated using the branching process (BP). Dots correspond to single runs of the model, crosses show the average value for the 10 independent simulations.

The first scenario tested corresponds to homeostasis in the progenitor population (*pp*−*dd*=0), constant value of *T*=20 h and no quiescent or apoptotic cells 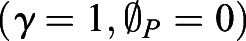. These are the conditions defined by Nowakowski and co-workers when introducing originally the cumulative curve method (Nowakowski et al., 1989). Results of the analysis are plotted in Fig. 5A. Dots in Fig. 5B correspond to the prediction of the value of *T* for 10 independent simulations (crosses represent the average). We see that, for these particular settings, all four methods are able to predict the correct value of *T* (dashed line) within a 10% error margin, with both PC and BP performing slightly better than C1 and C2. Importantly, when comparing the individual values for the 10 simulations predicted by single and double cumulative curve methods (C1 and C2), there is a higher dispersion than in PC and BP methods. This means that a high number of repeats should be necessary to obtain an accurate value of *T*, and that the typical experimental design that involves only three independent repeats does not guarantee a correct estimation of the cell cycle. The same conclusions apply when considering growth of the population of progenitors, as in the case of RG reported here (*pp*−*dd*>0; Fig. S4A).

Variable cell cycle dynamics have been reported in many developmental systems (Míguez, 2015; Saade et al., 2013; Le Dréau et al., 2014; Takahashi et al., 1995; Calegari and Huttner, 2003; Calegari et al., 2005; Dehay and Kennedy, 2007; Mairet-Coello et al., 2012; Roccio et al., 2013; Arai et al., 2011; Iulianella et al., 2008; Locker et al., 2006). Fig. 5C shows the output of the numerical model when a variable value of *T* is used as input (with an average value *T*=20 h). Fig. 5D plots the quantification of the cell cycle in these conditions. In this situation, C1 predicts a much longer cell cycle that the average (49% error), while the C2 predicts a shorter cell cycle (24% error). Interestingly both PC and BP return a value much closer to the correct average, with less than 10% error. Again, the variability of the single cumulative C1 method (the one used in Fig. 1E,F and the most commonly used in the literature) is very high, making it unreliable when a small number of repeats are used (less than 10). Again, the same conclusions apply when considering conditions where the cell cycle changes while the population of progenitors is allowed to grow (*pp*−*dd*>0; Fig. S4B).

The balance between differentiative and proliferative divisions has been shown to also change over time in many developmental systems (Saade et al., 2013; Míguez, 2015). We show here that, even *in vitro*, with cells growing in constant controlled conditions, the mode of divisions is highly non-constant (Fig. 3A). When we set a variable *pp−dd* in our simulations, we observe that, again, both single C1 and dual C2 cumulative methods fail and show high dispersion between independent samples (Fig. S4C). The same occurs when both mode and rate of division are allowed to change simultaneously (Fig. S4D). In these more realistic conditions closer to our experimental findings (variable mode and rate of division), the branching process equation predicts a value that is closer to the one used in the simulations, and the variability between samples is highly reduced.

In addition, our simulations show that the growth fraction measured with C1 method (the one used in Fig. 1) works well when the cell cycle and mode or division are constant, but it is not accurate when the parameters are variable. Therefore, the method based on Ki67 immunostaining (Fig. 4B) is more accurate under these conditions.

In conclusion, these results show that methods based on cumulative curve labeling are not suitable when proliferation and/or differentiation rates are not constant. This, together with the reported effect of BrdU and analogs in lengthening the cell cycle (Levkoff et al., 2008), and the high dispersion when comparing sets of cells growing under the same exact conditions, could explain the discrepancy values of the cell cycle reported in Figs 1E,F and 4C. In addition, the error in the growth fraction measured in Fig. 1E,F versus 4A can be due to the same problems. Both pulse-chase (PC) and branching process (BP) perform well, while the branching formalism has the advantage of providing temporal resolution, as well as accurate values of the average mode of division during the experiment.

### Values from the branching process analysis are able to reproduce the experimental data

To test whether the values provided by the branching process formalism are correct, we took advantage of the same numerical model of the differentiating stem cell population introduced previously. Now, the model is informed with the values of initial number of cells as in the experiments (Fig. 2B,C), the values of *T* and *pp*−*dd* (Figs 3A and 4C), and the growth fraction *γ* and apoptosis measured in the previous sections (Fig. 4B; Fig. S1B).

**Fig. 6.**
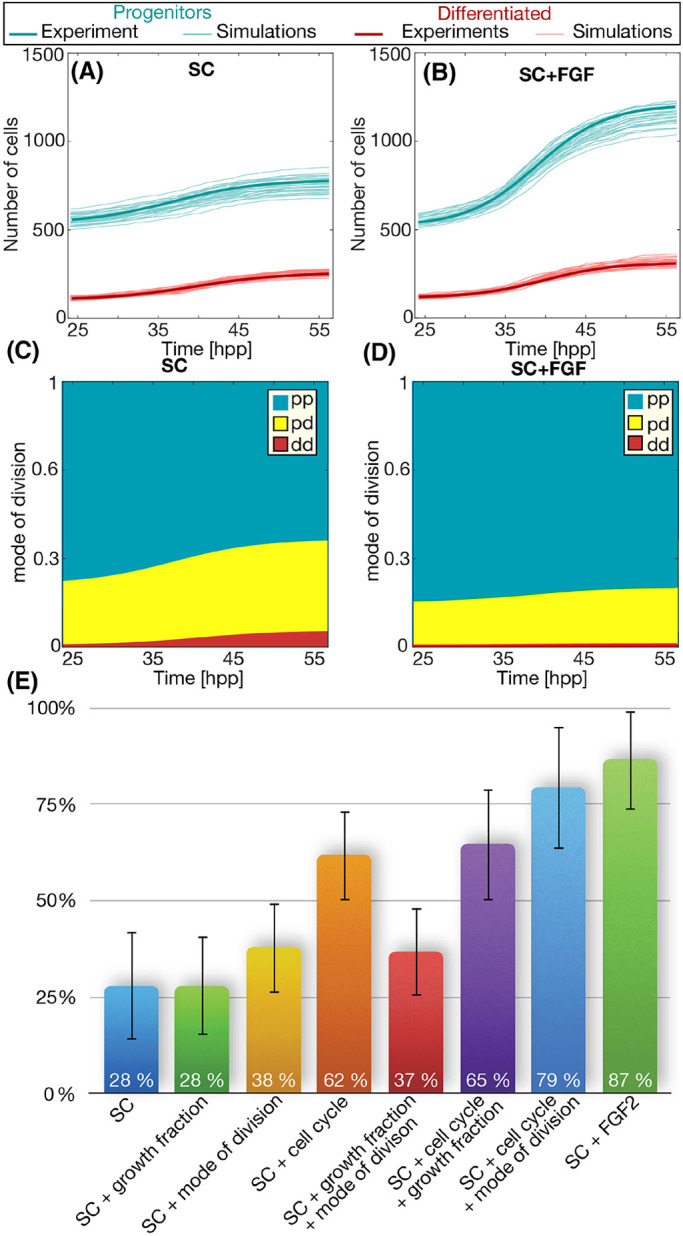
**Values derived using the branching process formalism reproduce the correct dynamics observed experimentally.** (A,B) Numerical simulations (light red and blue lines) for both conditions using the parameters of mode and rate of division predicted by the equations of the branching process. Thick lines correspond to the sigmoidal fitting of the experimental data in Fig. 2. (C,D) Prediction for the rate of each mode of division assuming a pure probabilistic scenario for the differentiation (i.e. the fate of the daughter cells independent of each other). (E) Changes in the population of cycling progenitors due to each of the three effects of FGF2 alone or in combination.

The model returns numbers of progenitors and differentiated cells that can be compared with the values of progenitors and differentiated cells measured experimentally. Accordingly, if simulations are able to reproduce the experimental observations, then the values of *T* and *pp*−*dd* obtained using the branching process formalism are correct. This comparison is shown in Fig. 6A,B, where the number of progenitors (thin blue lines) and differentiated cells (thin red lines) are plotted for 30 independent simulations using the same input values. Thick blue and red lines are the sigmoidal fittings of the experimental data. The good agreement between simulations and experiments under both conditions (SC and SC+FGF) suggests that the values of *T* and *pp*−*dd* predicted by the branching equations are correct.

## DISCUSSION

A detailed analysis of the dynamics of vertebrate neurogenesis involves a careful characterization of the features that regulate the dynamics of proliferation and differentiation of RG during the generation of the mammalian cortex. One of its most striking features is the fact that RG are restricted to an asymmetric mode of division *in vivo*, as opposed to a more probabilistic scenario observed in other developmental systems (Saade et al., 2013; Míguez, 2015; He et al., 2012; Chen et al., 2012; Clayton et al., 2007; Teixeira et al., 2013; Klein et al., 2010; Snippert et al., 2010). FGF2 has been shown to facilitate the expansion of RG *in vitro* cultures, but the details of this process have not been studied. Our quantitative characterization of the role of FGF2 shows simultaneous effects in the growth fraction (Fig. 4B), in the mode of division (Fig. 3A) and in the length of the cell cycle (Fig. 4C).

The overall influence of each of these effects in the expansion potential of the RG culture can be assessed using our numerical model. To do this, we informed the simulations with the experimental values for SC, and quantified the increase in the number of cycling progenitors (as a measure of the potential of the culture to expand in size) after 22 h. Next, we substituted each of the predictions for cell cycle length, growth fraction and differentiation rate predicted for the SC+FGF2 conditions, individually or in combination. The increase in cycling progenitors for 30 independent numerical simulations for each condition is shown in Fig. 6E. Surprisingly, the analysis suggests that the most influential feature is not the differentiation rate or the growth fraction, but the change in cell cycle length. The changes in growth fraction or the mode of division do not significantly impact the culture in terms of cycling progenitors (by 1% and 9%, respectively). On the other hand, the effect of mode of division combined with the cell cycle increases the expansion rate (51%), when compared with the effect of the cell cycle alone (34%).

Several authors propose that the mode of division depends on the distribution of cell fate determinants during mitosis, the orientation of the spindle or the inheritance of the primary cilium or the different centrosomes (Taverna et al., 2014). It is possible that the apical-basal polarized structure of the RG, or their organization and orientation of the radial processes along the stratified neuroepithelium results in asymmetric inheritance of these cell fate regulators (Taverna et al., 2014). The loss of these polarizing features provided by the niche when cells are cultured *in vitro* may result in a probabilistic scenario where the fate of the two daughter cells is independent of each other and all three modes of divisions are possible, similar to neuronal progenitor cells and other developmental systems (Saade et al., 2013; Míguez, 2015; He et al., 2012; Chen et al., 2012; Clayton et al., 2007; Teixeira et al., 2013; Klein et al., 2010; Snippert et al., 2010). In fact, early studies in the mouse neocortex suggest that the model that best fits the clone distribution assumes that the fates of the daughter cells are independent of each other (Cai et al., 2002). In this situation, the branching process framework is able to estimate the rates of each of the three modes of division (Míguez, 2015). This prediction for the case of RG in culture is shown in Fig. 6C,D, where we can see that the predominant mode of division is *pp* (green). This symmetric mode of division is even more probable under conditions of SC+FGF, at the expense of reductions in *pd* and *dd*.

A detailed analysis of the dynamics of vertebrate neurogenesis involves a careful characterization of the rate of division. The most-direct method to measure the cell cycle length requires the monitoring of the time between consecutive mitotic events at single cell resolution (Sigal et al., 2006; Wilcock et al., 2007). Unfortunately, owing to the high degree of variability, many cells in a population need to be sampled, segmented and tracked simultaneously to obtain an accurate value, even when dealing with clonal samples (Sandler et al., 2015). Therefore, the most-used approach is that of thymidine analogs, but this has several drawbacks: it can be toxic and mutagenic (Duque and Gorfinkiel, 2016), and can affect the normal dynamics of cell proliferation (Levkoff et al., 2008) by lengthening the cell cycle. In addition, choosing the correct mathematical analysis and interpretation of the experimental data is not straightforward (Johansson et al., 1999). Authors have proposed several approaches, such as linear fitting (Begg et al., 1985; Høyer et al., 1994), nonlinear fitting (Johansson et al., 1994; Weber et al., 2014), or the use of deterministic (Lee and Perelson, 2008) or stochastic models (Zilman et al., 2010). Depending on the method used, the same input data results in very different predictions for the average duration of the cell cycle (Ritter et al., 1992). Owing to these limitations, BrdU and analogs have been referred to as ‘one of the most misused techniques in neuroscience’ (Taupin, 2007).

Our results show that methods based on cumulative incorporation of thymidine analogs perform well in conditions of constant proliferation and differentiation, but they are not designed to study systems where the cell cycle changes over time, which is potentially the case in many developmental systems. Under these conditions, the branching process formalism and the pulse-chase outperform cumulative curve methods. On the other hand, the pulse-chase method requires experiments that are longer than the cell cycle length, so an estimation of the value of the cell cycle has to be known beforehand. In addition, the well-known toxic effect of the labeling agent for such long periods of time may affect strongly the normal cell cycle progression, probably by enlarging its real value (Levkoff et al., 2008; Duque and Gorfinkiel, 2016). A clear advantage of the branching process is that it does not involve manipulation of the samples before fixation, so there is no interference with the normal progression of the cell cycle. In addition, the branching process formalism also provides the correct value of *T* with temporal resolution and the measurement of the average differentiation rate (also with temporal resolution). Some of the more important limitations of the branching process tool are that it requires absolute values of numbers of cells and not relative amounts (thus, data from flow cytometry is not useful). In addition, it can only be used in situations where differentiated cells are terminally differentiated.

Several studies have shown that the length of G1 phase increases progressively when neurogenesis starts, resulting in a overall increase in cell cycle length (Takahashi et al., 1995; Calegari and Huttner, 2003; Calegari et al., 2005; Dehay and Kennedy, 2007; Mairet-Coello et al., 2012; Roccio et al., 2013). Alternatively, other studies show that the cell cycle length is shorter in neurogenic divisions, compared with proliferative divisions (Arai et al., 2011; Saade et al., 2013; Le Dréau et al., 2014; Iulianella et al., 2008; Locker et al., 2006), owing mainly to a shorter S phase. The potential effects of FGF2 in each phase of the cell cycle has been studied previously (Hitomi and Stacey, 1999). Our results show that FGF promotes *pp* divisions and shortens the cell cycle, consistent with the findings of Lukaszewicz et al. (2002), and with the hypothesis that proliferative divisions have a shorter cell cycle, maybe due to a shorter G1 phase, similar to the effect of insulin-like growth factor (Mairet-Coello et al., 2009; Hodge et al., 2004).

Based on this, two of the consequences of FGF stimulation (shortening *T* and inducing *pp* divisions) may correspond to two aspects of the same role of FGF. For example, FGF induces *pp* divisions that have been shown to be faster than neurogenic divisions (Calegari et al., 2005). On the other hand, the lengthening of the neuroepithelial cell cycle has been shown to be sufficient to trigger differentiation. Based on this ‘cell cycle length hypothesis’ (Calegari and Huttner, 2003), the shortening of the cell cycle mediated by FGF may result in more *pp* divisions. The link between cell cycle length and mode of division is a very interesting topic, and our branching process tool is a powerful tool with which to study this interplay (Míguez, 2015).

Another potential explanation of our observations takes into account the mutual inhibitor link between FGF and retinoid signaling (del Corral and Storey, 2004). As retinoid signaling promotes the expression of inhibitors of CDKs, as well as neurogenic genes, its repression by increased levels of FGF can explain the triple effect of FGF in promoting proliferation, accelerating the cell cycle and even increasing the growth fraction.

An additional physiological interpretation of our observations involves the reported ventralizing potential of FGF (Pilz et al., 2013). It has been shown that FGF drives expression of proteins and transcription factors typical of a ventral radial glial identity, such as progenitor cells in the developing ganglionic eminence. Here, a large number of sub-apical progenitors show self-renewing capabilities, and their cell cycle shortens with each generation. The fact that FGF results in cell cycle acceleration and more self-renewing divisions is consistent with the ventralizing potential of FGF.

In addition, previous studies in chick spinal cord have shown that FGF has an heterogeneous effect, inducing incomplete interkinetic nuclear migration and fast non-apical divisions in a subset of progenitors. Unfortunately, our approach based on fixed samples and immunofluorescence does not allow us to monitor individual cellular responses to investigate potential heterogeneous responses in the pool of RG.

Another interesting approach is to study the interplay between the two key signals (FGF and EGF) that have been shown to be key to establish the self-renewing expanding RG culture (Conti et al., 2005). A currently ongoing project of our lab is to take advantage of the framework used here (*in vitro* culture, automated image analysis, modeling and branching processes) to establish how FGF and EGF interplay to fully inhibit differentiation and promote self-renewing divisions in tissue cultures of RG (Conti et al., 2005).

### Conclusions

The culture and differentiation of RG cells *in vitro* provides a very good framework to study basic features that orchestrate the formation of the mammalian neocortex. In brief, the system provides a well-controlled environment where the effect of signaling molecules and other conditions can be tested reliably, while providing easier manipulation and imaging compared with studies performed *in vivo*. We use this framework to study the features that promote the expansion of RG in culture driven by FGF2. Our combined experimental/computational/theoretical approach can be also used to test the effect of other signaling networks by quantifying the cell cycle and mode of division after ligand stimulation or small molecule inhibition, after a comparison with a control culture.

## MATERIALS AND METHODS

### Preparation and culture of dissociated mouse cortical RG

Cells were obtained from mouse embryos of the C57 BL/6JRCC line at E11/E11.5, following standard methods described previously (see Hilgenberg and Smith, 2007). The initial time point is labeled as 0 h post-plating (hpp) and it is used as the reference point for our experiments. Briefly, after careful removing of the meninges, the cortex was isolated and placed in Hank's Buffered Salt Solution free of Ca^2+^ and Mg^2+^ (HBSS, ThermoFisher 14185). Next, samples were mechanically disaggregated using Pasteur pipettes and plated in coverslips treated with nitric acid and fibronectin at 10 µg/ml (Fisher Scientific; 15602707) to facilitate cell adhesion. Cells were plated at constant density (250,000 cells in each M24 well) for all experiments in Neurobasal medium without L-glutamine (ThermoFisher 21103-049), Glutamax (ThermoFisher 35050-038), B-27 (ThermoFisher 17504-044), penicillin, streptomycin and anti-mycotic (concentrations standard for cell culture). Media were complemented with 0.02 ng/µl of recombinant murine EGF (PeproTech 315-09, lot number 0517179-1) and 0.02 ng/µl of human basic FGF (PeproTech 100-18B, lot number 0311706-1). This culture medium is referred to as the standard culture (SC) condition in our study. Cells are allowed to rest for 1 day in the incubator to recover from the dissection process. At 24 hpp, the culture medium was changed for fresh SC media or for SC media complemented with additional human basic FGF at a final concentration of 0.06 ng/µl. This culture condition is labeled as SC+FGF in this study. All experimental protocols were carried out in accordance with the guidelines of the European Communities Directive (2012/63UE) and Spanish legislation (RD 53/2013).

### Immunofluorescence

Cells were fixed for 20 min at room temperature in 4% paraformaldehyde and washed twice for 5 min with 1× phosphate-buffered saline (PBS). Fixed cells are incubated with the permeabilization solution composed of Triton X-100 (ChemSupply 9002-93-1) at 0.6% in PBS 1× for 20 min at room temperature. Next, cells were washed three times with 1×PBS and blocking solution was added (bovine serum albumin, BSA; Sigma, A7906) at 3% in 1×PBS for at least 30 min. Later, cells were incubated with primary antibodies dissolved in the blocking solution overnight at 4°C. The next day, cells were washed with PBS three or four times for 5 min and incubated with secondary antibodies in the blocking solution for 45 min at room temperature, protected from light. Next, secondary antibodies were washed out (1×PBS three or four times for 5 min) and nuclei were stained with Hoechst 3342 (1/2000, ThermoFisher 1399) dissolved in 1×PBS for 5 min at room temperature. Finally, cells were washed in PBS, double distilled water and 70% ethanol. Cover-slips were finally mounted with Fluoromount G (Southern Biotechnology Associates, 0100-01) on microscope glass slides. Primary antibodies used were: anti-Sox2 (1/2000, GeneTex GTX124477), anti-Map2 (1/200, Santa Cruz Biotechnology sc-74421), anti-Pax6 (1/1000, BioLegend B244573); anti-cleaved caspase 3 (1/1000, Cell Signaling 9661) and anti-KI67 (1/200, ThermoFisher 14-5698-82). Secondary antibodies used were: anti-rabbit 488 (1/1000, ThermoFisher A-21206), anti-mouse 555 (1/1000, ThermoFisher A-21137) and anti-Rat 555 (1/1000, ThermoFisher A-21434).

### Statistical and data analysis

One-way ANOVA test was used to measure statistical significance between different time points. Cell cycle values in Fig. 1E,F were obtained after linear regression of the four first data points. Rates of quiescence in Fig. 4B were obtained from the mean value of the four last points. Slope error was calculated using a linear fitting with values of the average plus standard error and another linear fitting with values of the average minus standard error to obtain the difference in the slope between these two values. Quiescence error was the standard error of the four last points, and the *T* error was derived from the error propagation of the previous values. Three-parameter sigmoidal fitting was used to fit data from Fig. 2B,C. Sample size for all experiments was at least four. Unless specified, error bars represent the standard error of the mean, calculated using the error propagation. Confidence intervals for the prediction of *T* and *pp*−*dd* in Figs 3A and 4C were calculated by taking half of the value of the maximum error for *P* and *D* in Fig. 2B,C. Next, we fitted these data points to three-parameter sigmoidal functions. Finally, these curves were used as inputs to the branching process equations to obtain the 50% confidence intervals plotted. All curve fitting and statistical analysis were performed using Matlab (The Mathworks) and Julia programming language (Statistics package).

### EdU cumulative curve

A cumulative curve of the thymidine analog 5-ethynyl-2′-deoxyuridine (EdU) incorporation was created using a Click-iT Plus EdU Alexa Fluor 647 Imaging Kit (ThermoFisher, C10640). Briefly, EdU was added around 24 hpp at 2 μM. Cells were then fixed at increasing times of EdU exposition. Staining of EdU-positive cells was performed based on previously published protocols (Harrison et al., 2018). Immunostaining for Sox2 was used as a standard marker for RG progenitors (Beattie and Hippenmeyer, 2017). Later, the number of cells positive for both Sox2 and EdU was quantified using automated image processing. To calculate the cell cycle length, the percentage of progenitor cells that have incorporated EdU was plotted against the hours of EdU incorporation. The saturation value at long incubation times was used to calculate the growth fraction *γ*. This value was then used to calculate the average cell cycle using linear regression at short EdU accumulation times (see Fig. 1).

### EdU pulse-and-chase experiments

Cells were exposed to a short pulse of 30 min of EdU at 36 hpp. ‘Pulse’ points were fixed at this time point. ‘Chase’ points were washed three times with fresh medium and were fixed 15 h after the ‘Pulse’ time point. The number of EdU-positive/Sox2-positive cells is quantified for both ‘Pulse’ and ‘Chase’ time points for both conditions using automated image processing.

### Branching process formalism

Our lab has developed a method to measure the dynamics of proliferation and differentiation that does not depend on cumulative labeling with thymidine. Instead, it uses a branching process formalism, where cell cycle, growth fraction and mode of division are defined as independent variables, to derive analytical equations that provide the average values of proliferation and differentiation of the population based only on the numbers of proliferative, differentiated, quiescent and apoptotic cells at different time points. A scheme of the method is shown in Fig. S3D, and an example of its experimental implementation can be found in Míguez (2015). In brief, the branching process tool works with a minimum of two time points, and it does not require data points to be equispaced in time. In terms of repeats, depending on the interests of the works, it would be more useful to include more repeats to obtain more-reliable values, or more data points to obtain a more-detailed time evolution of the values. The number of data points needed to obtain a reliable temporal evolution depends on how fast cell numbers change (as a rule of thumb, we suggest a minimum of five time points each time the number of total cells doubles). In brief, if the user has a series of measurements of *P* and *D* (*n* experiments) for different time points (*t*_0_, *t*_1_, …, *t*_*m*_), they can simply use directly the mean of these data points as input of the equations to obtain discrete values of proliferation and differentiation rates between the time points. Another option is to fit the experimental values (using the mean and the standard deviation) to a representative polynomial curve (as we do here) and obtain a more smooth time evolution of the proliferation and differentiation rates.

To obtain these values, samples are allowed to develop without interfering with the normal dynamics of the cells, and then are fixed at different developmental times. After fixation, the amount of cells in each state is quantified by antibody staining to distinguish progenitors (*P*) (Graham et al., 2003), differentiated (*D*) (Míguez, 2013) and the number of progenitors undergoing apoptosis (Ø_*P*_) (Blanchard et al., 2010). The growth fraction *γ* is obtained using double immuno-labeling against Sox2 and Ki67 (Scholzen and Gerdes, 2000).

These values (quantified using the automated quantification described) are then fitted to sigmoidal functions (green and red curves in Fig. 2B,C) that are used as inputs of the following equations for the mode and rate of division, which correspond to a generalization of equations presented by Míguez (2015) updated to account for a potential reduction of the progenitor pool:
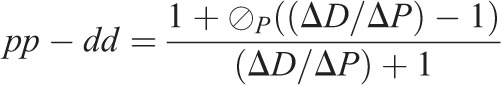

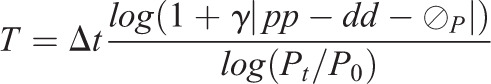
where *pp* and *dd* correspond to the rate of symmetric proliferative and differentiative divisions, respectively. Δ*P*=*P*_*t*_−*P*_0_ and Δ*D*=*D*_*t*_−*D*_0_ correspond to the number of progenitors and differentiated cells generated in a given window of time Δ*t*=*t*−*t*_0_. The value *pp*−*dd* goes from 1 (all divisions are symmetric proliferative) to −1 (all divisions are symmetric differentiative). The value, *pp*−*dd*=0 corresponds to maintenance of the progenitor pool, either via asymmetric *pd* divisions or via a balance between symmetric proliferative and differentiative divisions (the model cannot distinguish between these two scenarios, as they are mathematically equivalent). ∅_*P*_ is the rate of cell death of the progenitors pool, obtained using double immunolabeling against Sox2 and cleaved caspase 3 (see Fig. S1B). This reduced value of apoptosis rate (assuming that most cell death occurs via apoptosis) is consistent with estimations from *in vivo* experiments (Cai et al., 2002).

### Image acquisition and analysis

Samples were imaged using a confocal microscope (AR1+) that has a high speed of acquisition and sensibility coupled to an inverted microscope (Eclipse Ti-E, Nikon) with a 20× objective and a resolution of 1024×1024 pixels. The field of view was set to 0.6 mm×0.6 mm. In brief, image processing and analysis (performed in Fiji; Schindelin et al., 2012) was based on the segmentation of nuclei and the classification of each cell as progenitor, differentiated neuron, quiescent or apoptotic based on the intensity of the fluorescence staining of each marker. A large number of cells (around 10^5^ cells) was processed for each data point to minimize the effect of variability and heterogeneity of the samples. The sequence of processing algorithms and filters is as follows.

1. Definition of the Kernel Radius (*KR*) that sets the size of the region used for calculations and filter processing. Several *KR* sizes were tested (values from 1 to 5 pixels). The final *KR* was fixed as 2.5.
2. A local thresholding is applied to remove background based on the median intensity as cutoff value (radius=8×*KR*).
3. To remove breaks and holes inside the objects generated by the previous filter, the following sequence of filters is applied to enhance the definition of the boundaries of each object: Gaussian Blur filter, Maximum Filter, Median filter and Unsharp Mask filter (radius=KR).
4. The resulting image is binarized using the median value as threshold.
5. Euclidean distance mapping (EDT) is performed in the binary image to generate seeds that are used by a flood-fill algorithm to define the boundaries of each object (Kang et al., 2010).
6. Finally, all objects are fitted to ellipses for posterior analysis. Ellipses smaller than 4×*π*×*KR*^2^ are discarded from the analysis.

The specific features of each staining requires a different set of processing filters to enhance signal for each channel.

1. Map2: double sequential thresholding to extract foreground information (cutoff 1=mean, cutoff 2=median); morphological opening to remove neurons fibers (structuring element: lines at different angle with a length of 2×*KR*); Gaussian filter to remove noise (radius=KR).
2. Sox2: double sequential thresholding to extract foreground information (cutoff 1=mean, cutoff 2=median); morphological opening to select only nuclei with minimal size (structuring element: circumference of radius equal to 2×*KR*); Gaussian filter to remove noise (radius=KR).
3. EdU: single thresholding to extract foreground information (cutoff=median); morphological opening (structuring element: circumference of radius equal to 2×*KR*); Gaussian filter to remove noise (radius=KR).
4. Pax6: single thresholding to extract foreground information (cutoff=mean); morphological opening (structuring element: circumference of radius equal to 2×*KR*); Gaussian filter to remove noise (radius=KR).
5. Cleaved caspase 3: double sequential thresholding to extract foreground information (cutoff 1=mean, cutoff 2=mean+plus s.d.); morphological opening to select only nuclei with minimal size (structuring element: circumference of radius equal to 2×*KR*); Gaussian filter to remove noise (radius=KR).
6. Ki67: single thresholding to extract foreground information (cutoff=mean); morphological opening (structuring element: circumference of radius equal to 2×*KR*); Gaussian filter to remove noise (radius=KR).

Finally, the identity of each ellipse was established based on the number of pixels above threshold in each channel. For the Map2, this area was set to at least 15%; for the rest it was set to 1%. A subset of cells was both Sox2− and Map2−, and had a nucleus that was much larger that Sox2+ or Map2+. As these were not RG or differentiated neurons, they were not taken into account in the study.

### Numerical simulations of cell populations

We developed an *in silico* phenomenological numerical model of a generic differentiating stem cell population that simulates cycling progenitors that can either proliferate, differentiate, enter quiescence or enter apoptosis based on rates and probabilities provided by the user. Each cell has the following features: length of its cell cycle (*T*), current phase of cell cycle, time since birth (age) and fate (progenitor, quiescent, differentiated or apoptotic). Values of cell cycle length, mode of division, quiescence and death rate can be kept constant throughout the simulation or can be set to change each time-step. For each cell in the simulation, the cell cycle length, the probability of differentiation at the end of the cell cycle, the probability of entering quiescence and the probability of entering apoptosis are obtained from gamma distributed values with a mean value set by the input parameters (*T*, pp−dd, *γ*, and ∅_*P*_) and standard deviation of 30% of the mean to mimic intrinsic cell-to-cell variability and intrinsic noise in a clonal population (León et al., 2004) (other values of the standard deviation from 10% to 50% provide similar results).

A scheme of how the population is defined and develops over time is shown in Fig. S2. Parameters of the simulation are: the number of initial cells *m*, the average cell cycle *T* at each time point (defined as 
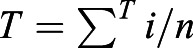
, *n* being the number of cells at time *t*), the fraction of cycling progenitors (or growth fraction) *γ*, the rate of apoptosis of progenitors ∅_*P*_ and the length of the experiment *t*_*end*_. The age of each cell is defined as the time since its birth, and the type corresponds to its characteristic as progenitors (*P*, cycling cells), differentiated (*D*, non cycling cells), quiescent (*Q*, non cycling progenitors) and apoptotic (dying cells).

The simulation takes palace as follows: an initial set of unsynchronized progenitor cells are allowed to cycle following the different phases of the cell cycle: from *G*_1_ to *S* *G*_2_ finally *M* phase. Upon division, the two resulting daughter cells either remain as progenitors (*pp* division), become terminally differentiated cells and stop cycling (*dd* division), or one remains as a progenitor while the other differentiates (*pd* division). For simplicity, the cell cycle is divided into just three main steps of equal length: *G*_1_, followed by *S* and finally followed by *G*_2_+*M* (*T*=*T*_*G*1_+*T*_*S*_+*T*_*G*2*M*_). Changes in the cell cycle length affect all phases of the cell cycle identically (simulations where the phases are of different length and where changes affecting different phases of the cell cycle in different ways show equivalent results).

### Simulations of cell cycle determination methods

The previous model is then adapted to perform a computational analog of one or two thymidine compounds. At any time in the simulation, EdU can be added to the cells, so cells undergoing S phase will be labeled as ‘positive’ and will remain as positive throughout the rest of the simulation. The input parameters of the model are varied to simulate different dynamics of a population of cells in different conditions, in terms of quiescence, apoptosis, cell cycle length and differentiation rate. For each condition tested, we perform four measurements of the cell cycle based on the following methodologies:

#### Cumulative curve method

This technique has been extensively used both in *in vitro* and *in vivo* situations to quantify the rate of cells in the population entering S phase (Martinez-Morales et al., 2010; Le Dréau et al., 2014). A scheme of the method is shown in Fig. S3A. In brief, a nucleoside analog is added to several identical samples that are fixed and stained at different times. Labeled cells in all samples are quantified using microscopy or flow cytometry. The ratio of progenitor cells that are labeled for each sample is plotted, and the values corresponding to the cell cycle length *T* are obtained from the slope of a linear regression fitting of the data at short exposure times. In addition, the fraction of cycling progenitor cells *γ*, or growth fraction, can be estimated from the rate of labeled cells after long exposure times. This method, when combined with dyes to measure DNA content, can be used to determine the length of the different phases of the cell cycle (Dolbeare and Selden, 1994).

#### Dual cumulative curve method

This method combines dual staining with thymidine analogs (Salic and Mitchison, 2008). It also provides the possibility of fixing all samples simultaneously to ensure that quantification is always performed at the same developmental time. In addition, it can also provide some positional information of regions in a given tissue where cells cycle at different rates (Shibui et al., 1989; Bradford and Clarke, 2011). However, it requires a more-complex experimental design, and it may also result in increased toxicity. In addition, it does not provide information about the growth fraction. The method (Fig. S3B) involves an initial labeling agent administered to all samples simultaneously, and a second agent administered at different time points. All samples are collected at the same time, and they are stained for both labeling agents. The amount of cells that are double positive over time for the two different thymine analogs is plotted, and the average length of *T* and *T*_*S*_ can be obtained using linear or nonlinear regression (some corrections regarding the potential differential incorporation of both agents are required).

#### Pulse-chase method

Both previous methods rely on long-term exposure of the samples to nucleoside analogs, which can result in toxicity effects. Alternative, a short pulse can also be applied (Weber et al., 2014) to label only cells that were in S phase at a given time. The population of positive cells is then ‘chased’ in the different samples by fixing and staining at different times. Several variations of this method have been developed. A commonly used technique is to stain cells in mitosis (using immunofluorescence against phospho-histone 3), or using a second thymine analog in S phase to chase cells that have re-entered in a new S phase. A scheme of the method is shown in Fig. S3C.

The ratio of double-positive cells in the different samples is plotted over time, and the average value of *T* corresponds to the time between the pulse and the maximum number of double-positive cells in the population. The slope of the curve at shorter time scales can be used to calculate the length of S phase. Measurements of the cell cycle using this method require significantly longer experiments than the two previous methods.

#### Branching process method

The number of cells and their fate as progenitor, differentiated, quiescent or apoptotic cells is recorded at each time point during the simulation. These values are then used as input of the branching process (Eqn 2). The average value is then plotted for each condition tested.

## Supplementary Material

Supplementary information

Reviewer comments

## References

[DEV189712C1] Alexiades M. R. and Cepko C. (1996). Quantitative analysis of proliferation and cell cycle length during development of the rat retina. *Dev. Dyn.* 205, 293-307. 10.1002/(SICI)1097-0177(199603)205:3<293::AID-AJA9>3.0.CO;2-D 8850565

[DEV189712C2] Arai Y., Pulvers J. N., Haffner C., Schilling B., Nüsslein I., Calegari F. and Huttner W. B. (2011). Neural stem and progenitor cells shorten s-phase on commitment to neuron production. *Nat. Commun.* 2, 154 10.1038/ncomms1155 21224845 PMC3105305

[DEV189712C3] Baguma-Nibasheka M., Macfarlane L. A. and Murphy P. R. (2012). Regulation of fibroblast growth factor-2 expression and cell cycle progression by an endogenous antisense RNA. *Genes* 3, 505-520. 10.3390/genes3030505 24704982 PMC3899992

[DEV189712C4] Beattie R. and Hippenmeyer S. (2017). Mechanisms of radial glia progenitor cell lineage progression. *FEBS Lett.* 591, 3993-4008. 10.1002/1873-3468.12906 29121403 PMC5765500

[DEV189712C5] Begg A. C., McNally N. J., Shrieve D. C. and Kärcher H. (1985). A method to measure the duration of DNA synthesis and the potential doubling time from a single sample. *Cytometry* 6, 620-626. 10.1002/cyto.990060618 4064842

[DEV189712C6] Blanchard G. B., Murugesu S., Adams R. J., Martinez-Arias A. and Gorfinkiel N. (2010). Cytoskeletal dynamics and supracellular organisation of cell shape fluctuations during dorsal closure. *Development* 137, 2743-2752. 10.1242/dev.045872 20663818

[DEV189712C7] Bradford J. A. and Clarke S. T. (2011). Dual-pulse labeling using 5-ethynyl-2′-deoxyuridine (EdU) and 5-bromo-2′-deoxyuridine (BrdU) in flow cytometry. In *Current Protocols in Cytometry* (ed. Robinson J. P. et al.), Wiley Online Library, Chapter 7, Unit 7.38 10.1002/0471142956.cy0738s55 21207361

[DEV189712C8] Buck S. B., Bradford J., Gee K. R., Agnew B. J., Clarke S. T. and Salic A. (2008). Detection of s-phase cell cycle progression using 5-ethynyl-2′-deoxyuridine incorporation with click chemistry, an alternative to using 5-bromo-2′-deoxyuridine antibodies. *BioTechniques* 44, 927-929. 10.2144/000112812 18533904

[DEV189712C9] Cai L., Hayes N. L., Takahashi T., Caviness V. S. and Nowakowski R. S. (2002). Size distribution of retrovirally marked lineages matches prediction from population measurements of cell cycle behavior. *J. Neurosci. Res.* 69, 731-744. 10.1002/jnr.10398 12205666

[DEV189712C10] Calegari F. and Huttner W. B. (2003). An inhibition of cyclin-dependent kinases that lengthens, but does not arrest, neuroepithelial cell cycle induces premature neurogenesis. *J. Cell Sci.* 116, 4947-4955. 10.1242/jcs.00825 14625388

[DEV189712C11] Calegari F., Haubensak W., Haffner C. and Huttner W. B. (2005). Selective lengthening of the cell cycle in the neurogenic subpopulation of neural progenitor cells during mouse brain development. *J. Neurosci.* 25, 6533-6538. 10.1523/JNEUROSCI.0778-05.2005 16014714 PMC6725437

[DEV189712C12] Chen Z., Li X. and Desplan C. (2012). Deterministic or stochastic choices in retinal neuron specification. *Neuron* 75, 739-742. 10.1016/j.neuron.2012.08.008 22958814 PMC3438524

[DEV189712C13] Clayton E., Doupé D. P., Klein A. M., Winton D. J., Simons B. D. and Jones P. H. (2007). A single type of progenitor cell maintains normal epidermis. *Nature* 446, 185-189. 10.1038/nature05574 17330052

[DEV189712C14] Conti L., Pollard S. M., Gorba T., Reitano E., Toselli M., Biella G., Sun Y., Sanzone S., Ying Q.-L., Cattaneo E. et al. (2005). Niche-independent symmetrical self-renewal of a mammalian tissue stem cell. *PLoS Biol.* 3, e283 10.1371/journal.pbio.0030283 16086633 PMC1184591

[DEV189712C15] Dehay C. and Kennedy H. (2007). Cell-cycle control and cortical development. *Nat. Rev. Neurosci.* 8, 438-450. 10.1038/nrn2097 17514197

[DEV189712C16] del Corral R. D. and Storey K. G. (2004). Opposing FGF and retinoid pathways: a signalling switch that controls differentiation and patterning onset in the extending vertebrate body axis. *Bioessays* 26, 857-869. 10.1002/bies.20080 15273988

[DEV189712C17] Dolbeare F. and Selden J. R. (1994). Immunochemical quantitation of bromodeoxyuridine: application to cell-cycle kinetics. *Methods Cell Biol.* 41, 297-316. 10.1016/S0091-679X(08)61724-0 7861968

[DEV189712C18] Dono R., Texido G., Dussel R., Ehmke H. and Zeller R. (1998). Impaired cerebral cortex development and blood pressure regulation in FGF-2-deficient mice. *EMBO J.* 17, 4213-4225. 10.1093/emboj/17.15.4213 9687490 PMC1170755

[DEV189712C19] Duque J. and Gorfinkiel N. (2016). Integration of actomyosin contractility with cell-cell adhesion during dorsal closure. *Development* 143, 4676-4686. 10.1242/dev.136127 27836966

[DEV189712C20] Elsen G. E., Bedogni F., Hodge R. D., Bammler T. K., MacDonald J. W., Lindtner S., Rubenstein J. L. R. and Hevner R. F. (2018). The epigenetic factor landscape of developing neocortex is regulated by transcription factors pax6→ tbr2→ tbr1. *Front. Neurosci.* 12, 571 10.3389/fnins.2018.00571 30186101 PMC6113890

[DEV189712C21] Englund C., Fink A., Lau C., Pham D., Daza R. A. M., Bulfone A., Kowalczyk T. and Hevner R. F. (2005). Pax6, tbr2, and tbr1 are expressed sequentially by radial glia, intermediate progenitor cells, and postmitotic neurons in developing neocortex. *J. Neurosci.* 25, 247-251. 10.1523/JNEUROSCI.2899-04.2005 15634788 PMC6725189

[DEV189712C22] Frederiksen K. and McKay R. D. (1988). Proliferation and differentiation of rat neuroepithelial precursor cells in vivo. *J. Neurosci.* 8, 1144-1151. 10.1523/JNEUROSCI.08-04-01144.1988 3357014 PMC6569254

[DEV189712C23] Gao P., Postiglione M. P., Krieger T. G., Hernandez L., Wang C., Han Z., Streicher C., Papusheva E., Insolera R., Chugh K. et al. (2014). Deterministic progenitor behavior and unitary production of neurons in the neocortex. *Cell* 159, 775-788. 10.1016/j.cell.2014.10.027 25417155 PMC4225456

[DEV189712C24] Graham V., Khudyakov J., Ellis P. and Pevny L. (2003). SOX2 functions to maintain neural progenitor identity. *Neuron* 39, 749-765. 10.1016/S0896-6273(03)00497-5 12948443

[DEV189712C25] Gritti A., Parati E. A., Cova L., Frolichsthal P., Galli R., Wanke E., Faravelli L., Morassutti D. J., Roisen F., Nickel D. D. et al. (1996). Multipotential stem cells from the adult mouse brain proliferate and self-renew in response to basic fibroblast growth factor. *J. Neurosci.* 16, 1091-1100. 10.1523/JNEUROSCI.16-03-01091.1996 8558238 PMC6578802

[DEV189712C26] Harrison H., Pegg H. J., Thompson J., Bates C. and Shore P. (2018). HIF1-alpha expressing cells induce a hypoxic-like response in neighbouring cancer cells. *BMC Cancer* 18, 674 10.1186/s12885-018-4577-1 29925335 PMC6011406

[DEV189712C27] Hartfuss E., Galli R., Heins N. and Götz M. (2001). Characterization of CNS precursor subtypes and radial glia. *Dev. Biol.* 229, 15-30. 10.1006/dbio.2000.9962 11133151

[DEV189712C28] He J., Zhang G., Almeida A. D., Cayouette M., Simons B. D. and Harris W. A. (2012). How variable clones build an invariant retina. *Neuron* 75, 786-798. 10.1016/j.neuron.2012.06.033 22958820 PMC3485567

[DEV189712C29] Hilgenberg L. G. W. and Smith M. A. (2007). Preparation of dissociated mouse cortical neuron cultures. *J. Vis. Exp.* 10, e562 10.3791/562 PMC2557074 18989405

[DEV189712C30] Hitomi M. and Stacey D. W. (1999). Cyclin d1 production in cycling cells depends on ras in a cell-cycle-specific manner. *Curr. Biol.* 9, 1075-1084. 10.1016/S0960-9822(99)80476-X 10531005

[DEV189712C31] Hodge R. D., D'Ercole A. J. and O'Kusky J. R. (2004). Insulin-like growth factor-i accelerates the cell cycle by decreasing g1 phase length and increases cell cycle reentry in the embryonic cerebral cortex. *J. Neurosci.* 24, 10201-10210. 10.1523/JNEUROSCI.3246-04.2004 15537892 PMC6730172

[DEV189712C32] Høyer M., Bentzen S. M., Salling L. N. and Overgaard J. (1994). Influence of sampling time on assessment of potential doubling time. *Cytometry* 16, 144-151. 10.1002/cyto.990160208 7924683

[DEV189712C33] Huttner W. B. and Kosodo Y. (2005). Symmetric versus asymmetric cell division during neurogenesis in the developing vertebrate central nervous system. *Curr. Opin. Cell Biol.* 17, 648-657. 10.1016/j.ceb.2005.10.005 16243506

[DEV189712C34] Hutton S. R. and Pevny L. H. (2011). SOX2 expression levels distinguish between neural progenitor populations of the developing dorsal telencephalon. *Dev. Biol.* 352, 40-47. 10.1016/j.ydbio.2011.01.015 21256837

[DEV189712C35] Iulianella A., Sharma M., Durnin M., Vanden Heuvel G. B. and Trainor P. A. (2008). Cux2 (cutl2) integrates neural progenitor development with cell-cycle progression during spinal cord neurogenesis. *Development* 135, 729-741. 10.1242/dev.013276 18223201 PMC3093760

[DEV189712C36] Johansson M. C., Baldetorp B., Bendahl P. O., Johansson R. and Oredsson S. M. (1994). An improved mathematical method to estimate DNA synthesis time of bromodeoxyuridine-labelled cells, using FCM-derived data. *Cell Prolif.* 27, 475-488. 10.1111/j.1365-2184.1994.tb01477.x

[DEV189712C37] Johansson C. B., Momma S., Clarke D. L., Risling M., Lendahl U. and Frisén J. (1999). Identification of a neural stem cell in the adult mammalian central nervous system. *Cell* 96, 25-34. 10.1016/S0092-8674(00)80956-3 9989494

[DEV189712C38] Juarez E. F., Lau R., Friedman S. H., Ghaffarizadeh A., Jonckheere E., Agus D. B., Mumenthaler S. M. and Macklin P. (2016). Quantifying differences in cell line population dynamics using CellPD. *BMC Syst. Biol.* 10, 92 10.1186/s12918-016-0337-5 27655224 PMC5031291

[DEV189712C39] Kang W. and Hébert J. M. (2015). FGF signaling is necessary for neurogenesis in young mice and sufficient to reverse its decline in old mice. *J. Neurosci.* 35, 10217-10223. 10.1523/JNEUROSCI.1469-15.2015 26180198 PMC4502262

[DEV189712C40] Kang W., Wong L. C., Shi S.-H. and Hébert J. M. (2009). The transition from radial glial to intermediate progenitor cell is inhibited by FGF signaling during corticogenesis. *J. Neurosci.* 29, 14571-14580. 10.1523/JNEUROSCI.3844-09.2009 19923290 PMC2826126

[DEV189712C41] Kang H., Lee S. H. and Lee J. (2010). Image segmentation based on fuzzy flood fill mean shift algorithm. In *2010 Annual Meeting of the North American Fuzzy Information Processing Society*, 10.1109/NAFIPS.2010.5548413.

[DEV189712C42] Klein A. M., Nakagawa T., Ichikawa R., Yoshida S. and Simons B. D. (2010). Mouse germ line stem cells undergo rapid and stochastic turnover. *Cell Stem Cell* 7, 214-224. 10.1016/j.stem.2010.05.017 20682447

[DEV189712C43] Kosodo Y., Röper K., Haubensak W., Marzesco A.-M., Corbeil D. and Huttner W. B. (2004). Asymmetric distribution of the apical plasma membrane during neurogenic divisions of mammalian neuroepithelial cells. *EMBO J.* 23, 2314-2324. 10.1038/sj.emboj.7600223 15141162 PMC419905

[DEV189712C44] Le Dréau G., Saade M., Gutiérrez-Vallejo I. and Martí E. (2014). The strength of SMAD1/5 activity determines the mode of stem cell division in the developing spinal cord. *J. Cell Biol.* 204, 591-605. 10.1083/jcb.201307031 24515346 PMC3926951

[DEV189712C45] Lee H. Y. and Perelson A. S. (2008). Modeling t cell proliferation and death in vitro based on labeling data: generalizations of the smith-martin cell cycle model. *Bull. Math. Biol.* 70, 21-44. 10.1007/s11538-007-9239-4 17701260

[DEV189712C46] León K., Faro J. and Carneiro J. (2004). A general mathematical framework to model generation structure in a population of asynchronously dividing cells. *J. Theor. Biol.* 229, 455-476. 10.1016/j.jtbi.2004.04.011 15246784

[DEV189712C47] Levkoff L. H., Marshall G. P., Ross H. H., Caldeira M., Reynolds B. A., Cakiroglu M., Mariani C. L., Streit W. J. and Laywell E. D. (2008). Bromodeoxyuridine inhibits cancer cell proliferation in vitro and in vivo. *Neoplasia* 10, 804-816. 10.1593/neo.08382 18680882 PMC2504767

[DEV189712C48] Locker M., Agathocleous M., Amato M. A., Parain K., Harris W. A. and Perron M. (2006). Hedgehog signaling and the retina: insights into the mechanisms controlling the proliferative properties of neural precursors. *Genes Dev.* 20, 3036-3048. 10.1101/gad.391106 17079690 PMC1620016

[DEV189712C49] Lodato S. and Arlotta P. (2015). Generating neuronal diversity in the mammalian cerebral cortex. *Annu. Rev. Cell Dev. Biol.* 31, 699-720. 10.1146/annurev-cellbio-100814-125353 26359774 PMC4778709

[DEV189712C50] Losick R. and Desplan C. (2008). Stochasticity and cell fate. *Science* 320, 65-68. 10.1126/science.1147888 18388284 PMC2605794

[DEV189712C51] Lukaszewicz A., Savatier P., Cortay V., Kennedy H. and Dehay C. (2002). Contrasting effects of basic fibroblast growth factor and neurotrophin 3 on cell cycle kinetics of mouse cortical stem cells. *J. Neurosci.* 22, 6610-6622. 10.1523/JNEUROSCI.22-15-06610.2002 12151540 PMC2001296

[DEV189712C52] Macdonald P. D. M. (1970). Statistical inference from the fraction labelled mitoses curve. *Biometrika* 57, 489-503. 10.1093/biomet/57.3.489

[DEV189712C53] Mairet-Coello G., Tury A. and DiCicco-Bloom E. (2009). Insulin-like growth factor-1 promotes G1/s cell cycle progression through bidirectional regulation of cyclins and cyclin-dependent kinase inhibitors via the phosphatidylinositol 3-kinase/akt pathway in developing rat cerebral cortex. *J. Neurosci.* 29, 775-788. 10.1523/JNEUROSCI.1700-08.2009 19158303 PMC3256126

[DEV189712C54] Mairet-Coello G., Tury A., Van Buskirk E., Robinson K., Genestine M. and DiCicco-Bloom E. (2012). p57(KIP2) regulates radial glia and intermediate precursor cell cycle dynamics and lower layer neurogenesis in developing cerebral cortex. *Development* 139, 475-487. 10.1242/dev.067314 22223678 PMC3252351

[DEV189712C55] Martinez-Morales P. L., Quiroga A. C., Barbas J. A. and Morales A. V. (2010). SOX5 controls cell cycle progression in neural progenitors by interfering with the WNT-β-catenin pathway. *EMBO Rep.* 11, 466-472. 10.1038/embor.2010.61 20448664 PMC2892326

[DEV189712C56] Matsuzaki F. and Shitamukai A. (2015). Cell division modes and cleavage planes of neural progenitors during mammalian cortical development. *Cold Spring Harbor Perspect. Biol.* 7, a015719 10.1101/cshperspect.a015719 PMC4563714 26330517

[DEV189712C57] Miyata T., Kawaguchi A., Okano H. and Ogawa M. (2001). Asymmetric inheritance of radial glial fibers by cortical neurons. *Neuron* 31, 727-741. 10.1016/S0896-6273(01)00420-2 11567613

[DEV189712C58] Molyneaux B. J., Arlotta P., Menezes J. R. L. and Macklis J. D. (2007). Neuronal subtype specification in the cerebral cortex. *Nat. Rev. Neurosci.* 8, 427-437. 10.1038/nrn2151 17514196

[DEV189712C59] Míguez D. G. (2013). Network nonlinearities in drug treatment. *Interdiscip. Sci. Comput. Life Sci.* 5, 85-94. 10.1007/s12539-013-0165-x 23740389

[DEV189712C60] Míguez D. G. (2015). A branching process to characterize the dynamics of stem cell differentiation. *Sci. Rep.* 5, 13265 10.1038/srep13265 26286123 PMC4541069

[DEV189712C61] Müller-Sieburg C. E., Cho R. H., Thoman M., Adkins B. and Sieburg H. B. (2002). Deterministic regulation of hematopoietic stem cell self-renewal and differentiation. *Blood* 100, 1302-1309. 10.1182/blood.V100.4.1302.h81602001302_1302_1309 12149211

[DEV189712C62] Noctor S. C., Martínez-Cerdeño V., Ivic L. and Kriegstein A. R. (2004). Cortical neurons arise in symmetric and asymmetric division zones and migrate through specific phases. *Nat. Neurosci.* 7, 136-144. 10.1038/nn1172 14703572

[DEV189712C63] Nowakowski R. S., Lewin S. B. and Miller M. W. (1989). Bromodeoxyuridine immunohistochemical determination of the lengths of the cell cycle and the DNA-synthetic phase for an anatomically defined population. *J. Neurocytol.* 18, 311-318. 10.1007/BF01190834 2746304

[DEV189712C64] Pilz G.-A., Shitamukai A., Reillo I., Pacary E., Schwausch J., Stahl R., Ninkovic J., Snippert H. J., Clevers H., Godinho L. et al. (2013). Amplification of progenitors in the mammalian telencephalon includes a new radial glial cell type. *Nat. Commun.* 4, 2125 10.1038/ncomms3125 23839311 PMC3717501

[DEV189712C65] Qian X., Davis A. A., Goderie S. K. and Temple S. (1997). FGF2 concentration regulates the generation of neurons and glia from multipotent cortical stem cells. *Neuron* 18, 81-93. 10.1016/S0896-6273(01)80048-9 9010207

[DEV189712C66] Raballo R., Rhee J., Lyn-Cook R., Leckman J. F., Schwartz M. L. and Vaccarino F. M. (2000). Basic fibroblast growth factor (fgf2) is necessary for cell proliferation and neurogenesis in the developing cerebral cortex. *J. Neurosci.* 20, 5012-5023. 10.1523/JNEUROSCI.20-13-05012.2000 10864959 PMC6772267

[DEV189712C67] Ritter M. A., Fowler J. F., Kim Y., Lindstrom M. J. and Kinsella T. J. (1992). Single biopsy, tumor kinetic analyses: A comparison of methods and an extension to shorter sampling intervals. *Int. J. Rad. Oncol. Biol. Phys.* 23, 811-820. 10.1016/0360-3016(92)90654-Z 1618673

[DEV189712C68] Roccio M., Schmitter D., Knobloch M., Okawa Y., Sage D. and Lutolf M. P. (2013). Predicting stem cell fate changes by differential cell cycle progression patterns. *Development* 140, 459-470. 10.1242/dev.086215 23193167

[DEV189712C69] Saade M., Gutiérrez-Vallejo I., Le Dréau G., Rabadán M. A., Miguez D. G., Buceta J. and Martí E. (2013). Sonic hedgehog signaling switches the mode of division in the developing nervous system. *Cell Rep.* 4, 492-503. 10.1016/j.celrep.2013.06.038 23891002

[DEV189712C70] Salic A. and Mitchison T. J. (2008). A chemical method for fast and sensitive detection of DNA synthesis in vivo. *Proc. Natl. Acad. Sci. USA* 105, 2415-2420. 10.1073/pnas.0712168105 18272492 PMC2268151

[DEV189712C71] Sandler O., Mizrahi S. P., Weiss N., Agam O., Simon I. and Balaban N. Q. (2015). Lineage correlations of single cell division time as a probe of cell-cycle dynamics. *Nature* 519, 468-471. 10.1038/nature14318 25762143

[DEV189712C72] Schindelin J., Arganda-Carreras I., Frise E., Kaynig V., Longair M., Pietzsch T., Preibisch S., Rueden C., Saalfeld S., Schmid B. et al. (2012). Fiji: an open-source platform for biological-image analysis. *Nat. Methods* 9, 676-682. 10.1038/nmeth.2019 22743772 PMC3855844

[DEV189712C73] Scholzen T. and Gerdes J. (2000). The ki-67 protein: from the known and the unknown. *J. Cell. Physiol.* 182, 311-322. 10.1002/(SICI)1097-4652(200003)182:3<311::AID-JCP1>3.0.CO;2-9 10653597

[DEV189712C74] Shibui S., Hoshino T., Vanderlaan M. and Gray J. W. (1989). Double labeling with iodo- and bromodeoxyuridine for cell kinetics studies. *J. Histochem. Cytochem.* 37, 1007-1011. 10.1177/37.7.2659659 2659659

[DEV189712C75] Sigal A., Milo R., Cohen A., Geva-Zatorsky N., Klein Y., Liron Y., Rosenfeld N., Danon T., Perzov N. and Alon U. (2006). Variability and memory of protein levels in human cells. *Nature* 444, 643-646. 10.1038/nature05316 17122776

[DEV189712C76] Snippert H. J., van der Flier L. G., Sato T., van Es J. H., van den Born M., Kroon-Veenboer C., Barker N., Klein A. M., van Rheenen J., Simons B. D. et al. (2010). Intestinal crypt homeostasis results from neutral competition between symmetrically dividing lgr5 stem cells. *Cell* 143, 134-144. 10.1016/j.cell.2010.09.016 20887898

[DEV189712C77] Suter D. M., Tirefort D., Julien S. and Krause K.-H. (2009). A sox1 to pax6 switch drives neuroectoderm to radial glia progression during differentiation of mouse embryonic stem cells. *Stem Cells* 27, 49-58. 10.1634/stemcells.2008-0319 18832594

[DEV189712C78] Takahashi M. (1966). Theoretical basis for cell cycle analysis I. Labelled mitosis wave method. *J. Theor. Biol.* 13, 202-211. 10.1016/0022-5193(66)90017-8 5647131

[DEV189712C79] Takahashi T., Nowakowski R. S. and Caviness V. S. (1995). The cell cycle of the pseudostratified ventricular epithelium of the embryonic murine cerebral wall. *J. Neurosci.* 15, 6046-6057. 10.1523/JNEUROSCI.15-09-06046.1995 7666188 PMC6577667

[DEV189712C80] Takahashi T., Nowakowski R. S. and Caviness V. S. (1996). The leaving or q fraction of the murine cerebral proliferative epithelium: a general model of neocortical neuronogenesis. *J. Neurosci.* 16, 6183-6196. 10.1523/JNEUROSCI.16-19-06183.1996 8815900 PMC6579174

[DEV189712C81] Taupin P. (2007). BrdU immunohistochemistry for studying adult neurogenesis: paradigms, pitfalls, limitations, and validation. *Brain Res. Rev.* 53, 198-214. 10.1016/j.brainresrev.2006.08.002 17020783

[DEV189712C82] Taverna E., Götz M. and Huttner W. B. (2014). The cell biology of neurogenesis: toward an understanding of the development and evolution of the neocortex. *Annu. Rev. Cell Dev. Biol.* 30, 465-502. 10.1146/annurev-cellbio-101011-155801 25000993

[DEV189712C83] Teixeira V. H., Nadarajan P., Graham T. A., Pipinikas C. P., Brown J. M., Falzon M., Nye E., Poulsom R., Lawrence D., Wright N. A. et al. (2013). Stochastic homeostasis in human airway epithelium is achieved by neutral competition of basal cell progenitors. *eLife* 2, e00966 10.7554/eLife.00966 24151545 PMC3804062

[DEV189712C84] Teles J., Pina C., Edén P., Ohlsson M., Enver T. and Peterson C. (2013). Transcriptional regulation of lineage commitment–a stochastic model of cell fate decisions. *PLoS Comput. Biol.* 9, e1003197 10.1371/journal.pcbi.1003197 23990771 PMC3749951

[DEV189712C85] Weber T. S., Jaehnert I., Schichor C., Or-Guil M. and Carneiro J. (2014). Quantifying the length and variance of the eukaryotic cell cycle phases by a stochastic model and dual nucleoside pulse labelling. *PLoS Comput. Biol.* 10, e1003616 10.1371/journal.pcbi.1003616 25058870 PMC4109856

[DEV189712C86] Wilcock A. C., Swedlow J. R. and Storey K. G. (2007). Mitotic spindle orientation distinguishes stem cell and terminal modes of neuron production in the early spinal cord. *Development* 134, 1943-1954. 10.1242/dev.002519 17470968 PMC7116174

[DEV189712C87] Zilman A., Ganusov V. V. and Perelson A. S. (2010). Stochastic models of lymphocyte proliferation and death. *PLoS ONE* 5, e12775 10.1371/journal.pone.0012775 20941358 PMC2948000

