# Supplementary material for: FGF2 modulates simultaneously the mode, the rate of division and the growth fraction in cultures of radial glia
Source: Development. 2020 Jul 24;147(14):dev189712. doi: 10.1242/dev.189712 (PMC7390635; doi:10.1242/dev.189712)
Supplement: Supplementary information [file develop-147-189712-s1.pdf]

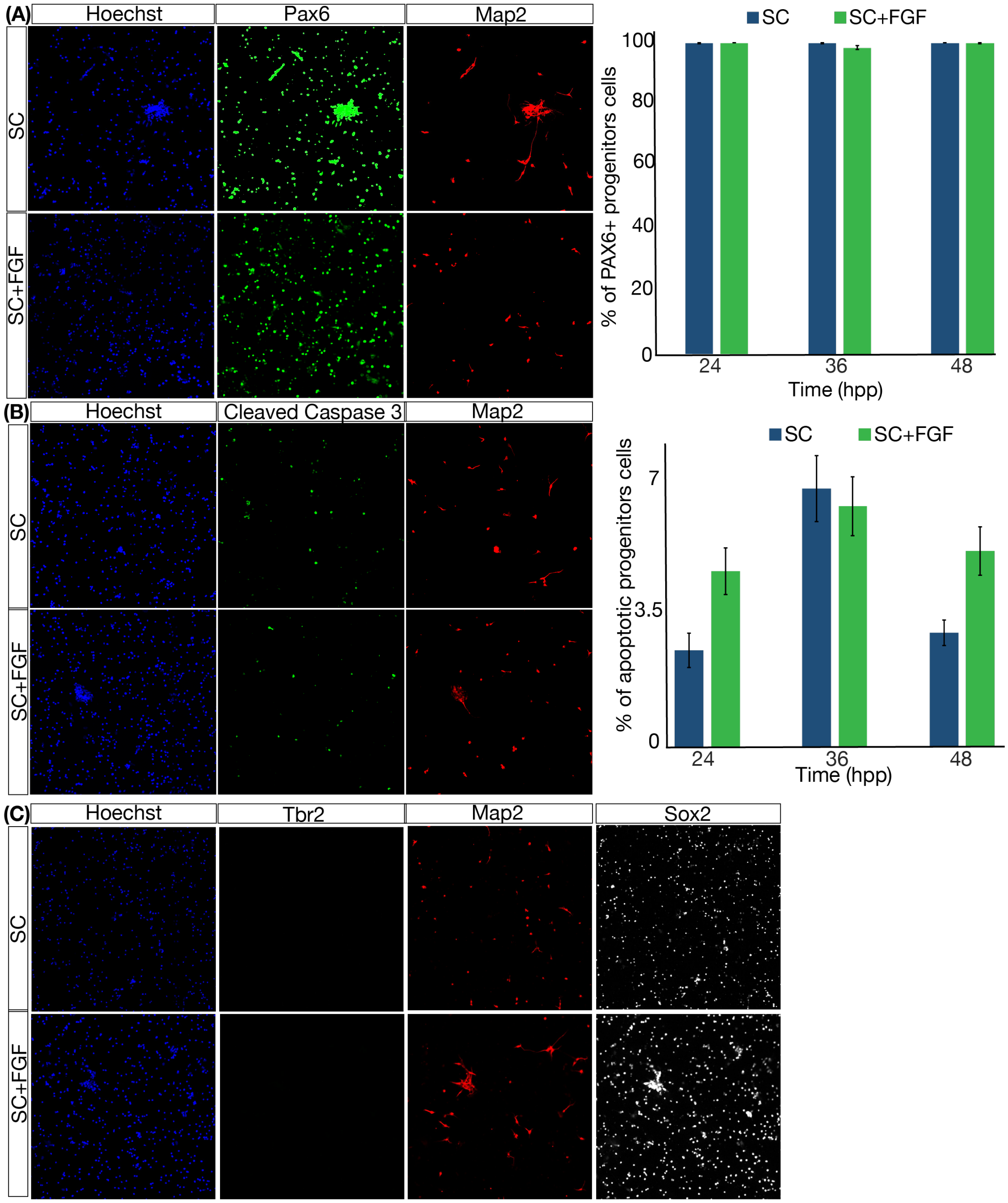

Figure S1: Experiments to obtain the apoptosis rate and the amount of NEP. (A) Example of cells stained with nuclei marker (blue), Pax6 (green), and Map2 (red) at 36 hpp. Quantification of the percentage of progenitors that are Pax6 positive for all conditions and three time points. Columns represent the mean between independent repeats. (B) Example of cells stained with nuclei (blue), cleaved Caspase3 (green), and Map2 (red) at 36 hpp. Quantification of the percentage of progenitor cells that show positive staining for Caspase3 for both conditions and at three different time points. This low value of apoptosis rate is consistent with estimations from *in vivo* experiments (Cai et al., 2002). (C) Immunostaining against Tbr2, Sox2 and Map2 (background level for Tbr2 was set as the intensity of green in the Map2+ (differentiated cells have been shown to be Tbr2- (Englund et al., 2005)). Error bars represent the standard error or the mean.

Development • Supplementary information

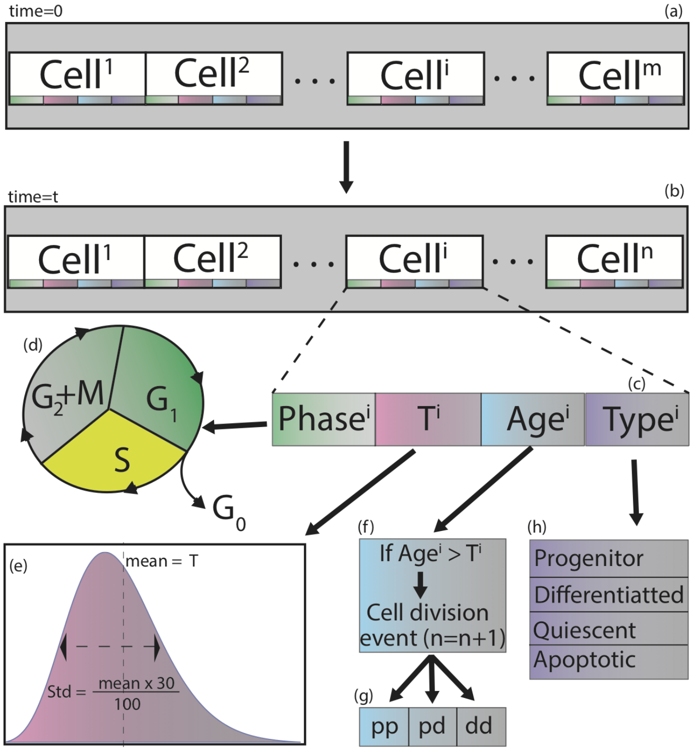

Figure S2: Scheme of the simulation of the population model. In brief: (a) an initial number m of un-syncronized progenitor cells proliferates and differentiates. (b) At any given time t, each cell i in the population of n cells is characterised by four parameters (c): phase, T, age and Type.(d) Cells cycle in their phase from G_1 to S to $G_2 + M$. When a given cell i reaches the end of $G_2 + M$, a division event takes place, with three different outcomes (g): pp, pd or dd division. In the presence of a labelling agent, cells incorporate it only during S-phase, and become labeled as "positive" (showed in yellow). Depending on their type, cells are sorted into 4 groups (h): progenitors, differentiated, quiescent and apoptotic.

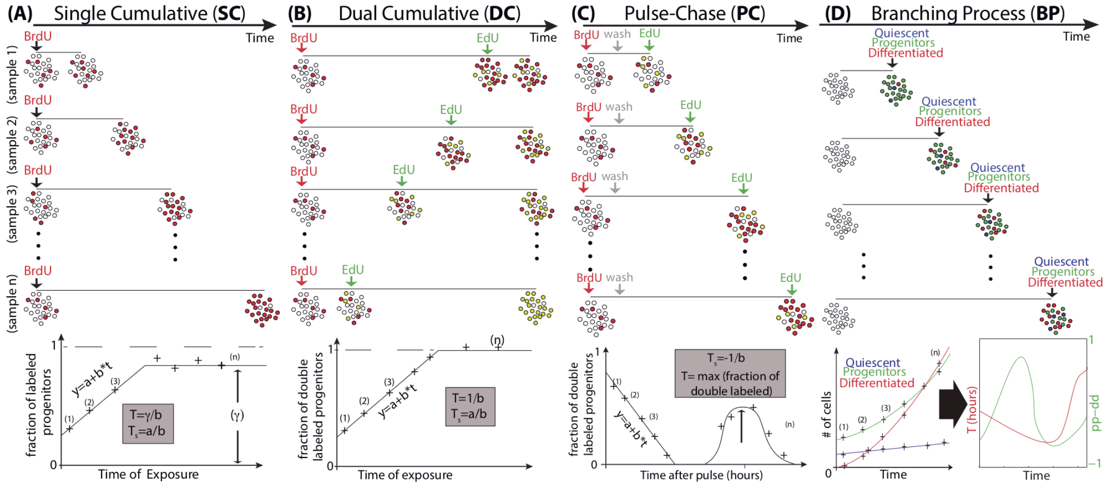

Figure S3: Scheme of methods tested to measure cell cycle length. (A) Cumulative curve: A thymidine analog is added to all samples simultaneously. Samples are fixed at different times and stained for quantification. Linear fitting of the rate of labelling is used to determine the average T and γ_P. (B) Dual Cumulative: The first thymidine analog (red) is administered to all samples simultaneously. The second thymine analog (green) is administered at different times. All samples are then fixed simultaneously. Quantification of all double positive cells (yellow) is plotted against exposure time. This method does not provide an estimation of the growth fraction. (C) Pulse-chase: A short pulse of a first nucleoside analog is added to all samples simultaneously. A second nucleoside analog is added at different times, and the samples are fixed and stained immediately after. The amount of double positive cells is plotted overtime. (D) Branching process: Cells are fixed at different times and stained with antibodies to distinguish progenitors, differentiated, quiescent and apoptotic cells. The resulting numbers are used to inform the equations **??-??**, that will give us the values of the average rate and mode of division overtime.

Development • Supplementary information

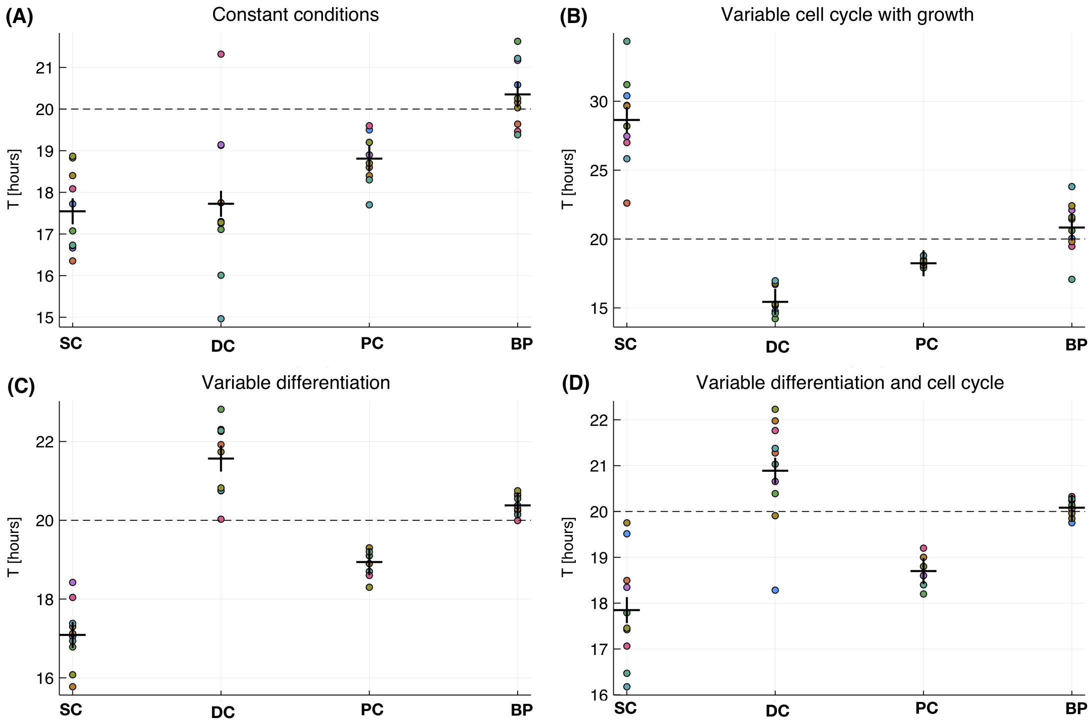

Figure S4: Values of cell cycle predicted for different conditions of growth and differentiation of the culture. Dots correspond to independent simulations. Crosses represent the average between 10 simulations. Dashed horizontal line corresponds to the average value of T used in the simulations (20 hours). Shorter distance between crosses and dashed line represent better performance of the method. Lower dispersion between dots in each method represents better accuracy. (A) Predicted value of T by each method in conditions of constant mode and rate of division, but for values of increased in the population of progenitors ($pp - dd > 0$). (B) Predicted value of T by each method in conditions where the cell cycle is set to decrease and then increase. (C) Predicted value of T by each method in conditions where the differentiation is increasing monotonically during the simulation. (D) Predicted value of T by each method in conditions where both cell cycle and differentiation rate are set to change during the simulation.

Development • Supplementary information

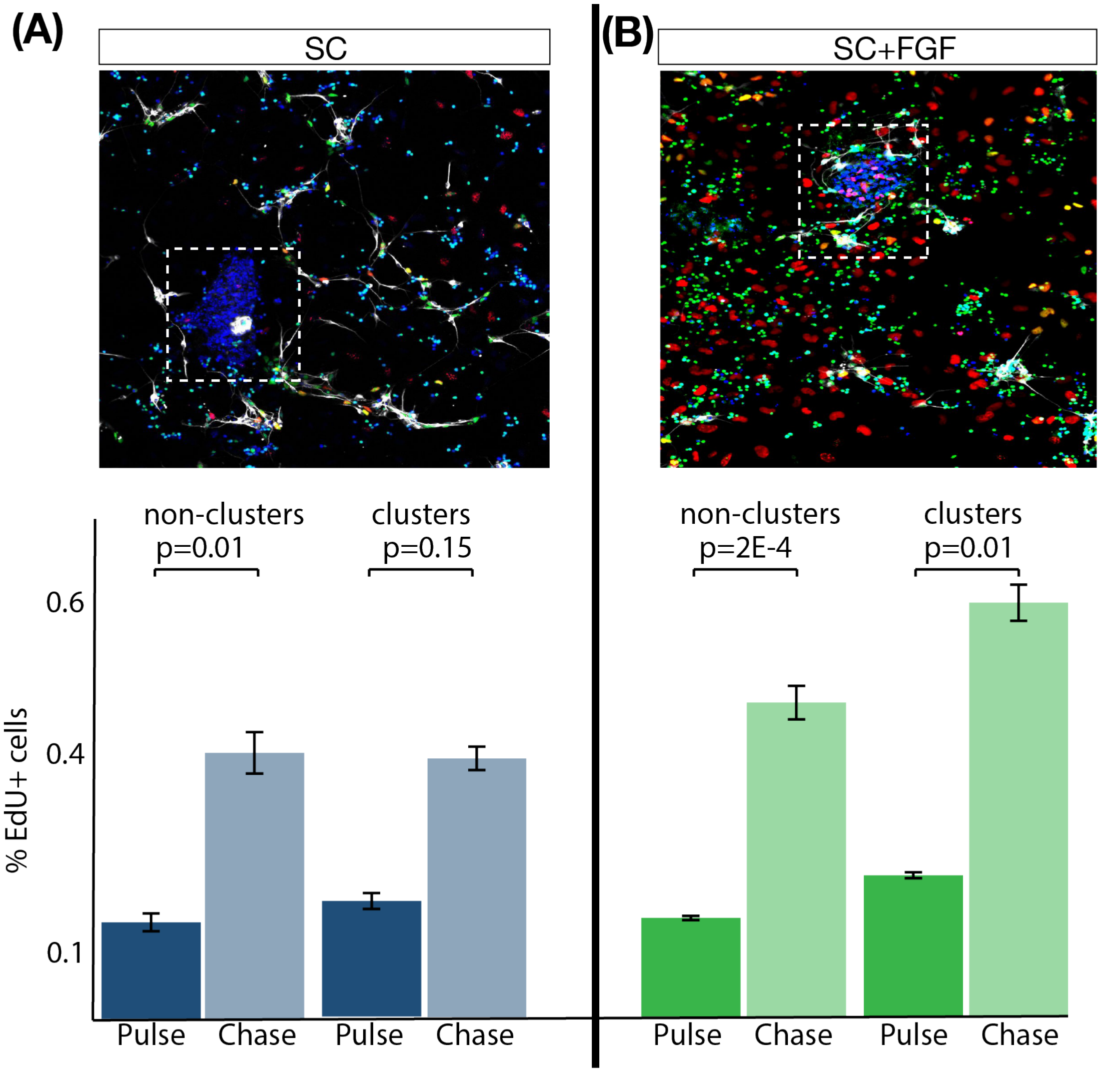

Figure S5: Heterogeneity in cell cycle length in clusters versus non cluster cells. (A-B) Representative snapshots of the culture where regions with cells organized in clusters are marked inside a white dashed square. Quantification of Edu+ cells in clusters and non clusters for the "Pulse" and "Chase" time points for (A) SC conditions and (B) SC+FGF conditions. Comparison of the increase in Edu+ cells between clusters and non-clusters suggests that cells cycle at a similar speed in both configurations.

Development • Supplementary information
