# Supplementary material for: FGF2 modulates simultaneously the mode, the rate of division and the growth fraction in cultures of radial glia
Source: Development. 2020 Jul 24;147(14):dev189712. doi: 10.1242/dev.189712 (PMC7390635; doi:10.1242/dev.189712)
Supplement: Reviewer comments [file develop-147-189712_review_history.pdf]

FGF2 modulates simultaneously the mode, the rate of division and the growth fraction in cultures of Radial Glia

Mario Ledesma-Terrón, Nuria Peralta-Cañadas and David G. Míguez
DOI: 10.1242/dev.189712

Editor: James Briscoe

Review timeline

Original submission:	6 March 2020
Editorial decision:	14 April 2020
First revision received:	27 April 2020
Editorial decision:	17 June 2020
Second revision received:	17 June 2020
Accepted:	18 June 2020

Original submission

First decision letter

MS ID#: DEVELOP/2020/189712

MS TITLE: FGF2 modulates simultaneously the mode, the rate of division and the growth fraction in cultures of Radial Glia

AUTHORS: Mario Ledesma-Terron, Nuria Peralta-Canadas, and David G Miguez

I have now received all the referees' reports on the above manuscript, and have reached a decision. The referees' comments are appended below, or you can access them online: please go to BenchPress and click on the 'Manuscripts with Decisions' queue in the Author Area.

As you will see, the referees express considerable interest in your work, but have some significant criticisms and recommend a substantial revision of your manuscript before we can consider publication. Each of the referees ask for specific clarifications that I think will improve the accessibility of your work for our readers. In addition, the referees have suggestions for strengthening the conclusions and for discussing the interpretation. In particular I would draw your attention to the comments from Referee 2 concerning expanding the description and discussion of the branching process model in the Methods and mentioning the approach in the Abstract. Referee 3 raises an important point about the possibility that FGF2 induces a ventral radial glial identity that result in cells adopting a symmetrically expanding neural progenitor identity typical of progenitors in the ganglionic eminence. In addition, this referee highlights the potential role of EGF. Ideally, these should be tested experimentally, however as this is unlikely to be feasible currently, the possibility should be acknowledged and implications considered in the Discussion. Referee 1 makes several helpful suggestions including asking whether it would be possible to explore in the model how the emergence of a population of progenitors lacking apical-basal polarity could help explain the observations

If you are able to revise the manuscript along the lines suggested, I will be happy to receive a revised version of the manuscript. Your revised paper will be re-reviewed by one or more of the original referees, and acceptance of your manuscript will depend on your addressing satisfactorily the reviewers' major concerns. Please also note that Development will normally permit only one round of major revision.

© 2020. Published by The Company of Biologists under the terms of the Creative Commons Attribution License (https://creativecommons.org/licenses/by/4.0/).

We are aware that you may currently be unable to access the lab to undertake experimental revisions. If it would be helpful, we encourage you to contact us to discuss your revision in greater detail. Please send us a point-by-point response indicating where you are able to address concerns raised (either experimentally or by changes to the text) and where you will not be able to do so within the normal timeframe of a revision. We will then provide further guidance. Please also note that we are happy to extend revision timeframes as necessary.

Please attend to all of the reviewers' comments and ensure that you clearly highlight all changes made in the revised manuscript. Please avoid using 'Tracked changes' in Word files as these are lost in PDF conversion. I should be grateful if you would also provide a point-by-point response detailing how you have dealt with the points raised by the reviewers in the 'Response to Reviewers' box. If you do not agree with any of their criticisms or suggestions please explain clearly why this is so.

Reviewer 1

Advance summary and potential significance to field

This paper presents a thoughtful analysis of the effects of FGF signalling on neural progenitor (radial glia) cell behaviour in vitro. The authors take a fresh approach (using their previously published Branched process tool) to measure and model changes that take place in conditions of increased FGF exposure. In particular, they demonstrate variability associated with calculations of growth fraction and cell cycle length when cumulative labelling approaches are used to determine these values and show greater reproducibility when pulse chase or the branched process approach is applied. This is a useful new analysis of a well-studied phenomenon, some comments for improving the paper are provided below.

Comments for the author

1) It would be useful to mention the differences found between data generated by distinct cell labelling methods and subsequent analyses in the Abstract. This is a strength of the paper which should be emphasised.

2) Key findings presented in the paper are that increased exposure to FGF alters cell division mode (more PP divisions) and that the cell cycle is shortened. This is nicely demonstrated using data on numbers of cells in distinct states identified by immunocytochemistry. Earlier work showing that RG cells accelerate the cell cycle in response to FGF has been published by the Dehay group and should be acknowledged and discussed Lukaszewicz, A. et al 2002 J. Neurosci. 22, 6610-6622.

3) As the authors note, another way in which such changes can be studied is to monitor cell behaviour in real time and some relevant work on this has been missed too (Wilcock A. et al 2007 Development 134, 1943-1954). In particular this paper shows that the response of neuroepithelium to FGF exposure is heterogenous: not only is the cell cycle time reduced and more PP divisions observed, but there is an increase in non-apical divisions (which have even shorter cell cycle times) and a breakdown of interkinetic nuclear migration in a subset of cells.
The possibility of heterogeneity in response to FGF should be discussed by the authors here – this may relate to differences in the action of FGF in different phases of the cell cycle, as reported by Hitomi, M. and Stacey, D. W. (1999) Curr. Biol. 9, 1075-1084.

4) A survey of apico-basal polarity markers would be further informative of the effects of RG exposure to increased FGF in vitro. Antibodies for these are readily available, for example those for N-Cadherin, aPKC, work well. This might reveal whether some or all cells in vitro are behaving in the same way. For example, images for Sox2 in Figure 2A cells in the SC condition are forming a small rosette, which is likely to be largely polarised cells, but the more extensive cell group in the SC+ FGF condition may include cells outwith rosettes lacking A/B polarity which may equate to the faster dividing cells? Given current constraints on laboratory working would it be possible to model heterogeneity using an estimated size of a potential A/B polarity lacking cell population in the increased FGF condition? Could the reduced cell cycle time be ascribed to emergence of this cell population over time?

© 2020. Published by The Company of Biologists under the terms of the Creative Commons Attribution License (https://creativecommons.org/licenses/by/4.0/).

5) Given the effect of FGF on cell cycle time some more consideration/discussion of the longer cell cycle that is characteristic of asymmetrically dividing neural progenitors should be provided. The known ability of FGF to repress retinoid signalling which is required for expression of cell cycle genes including cdk inhibitors and for expression of neurogenic genes and ultimately neuronal differentiation should also be discussed when considering how FGF influences proliferation.

Minor comments

1) Would be good to include the Tbr2 data, which is currently not shown. Tbr2 expressing cells are transit amplifying progenitors which undergo symmetric divisions and ultimately undergo division to generate two neurons. It is important to distinguish between these and NE cells that only divide to produce proliferative progeny.

2) Reconsider colours in 4B (blues too similar) and 6A-D, avoiding red/green.

Reviewer 2

Advance summary and potential significance to field

The manuscript “FGF2 modulates simultaneously the mode, the rate of division and the growth fraction in cultures of Radial Glia” investigates how FGF2 affects the differentiation dynamics of Radial Glial progenitors in the in vitro self-renewing culture. By combining experimental and numerical methods the FGF2 is identified to shorten the average cell cycle, promote progenitor-progenitor divisions and increase the growth fraction in the differentiation dynamics of Radial Glia in vitro. The population model of proliferating and differentiating cells is used to compare accuracy of four methods for estimating the cell cycle length. The Branching Process (BP) method is found to provide the most accurate cell cycle estimate among the four considered methods.

The proposed BP method is based on a stochastic formalisms that allows to make predictions about temporal changes in parameters describing growing population of cells. The BP method itself is not restricted to in vitro systems and can be potentially applied to many other in vivo developmental systems. Thus the BP method might be of high interest for stem cell and developmental biology communities, as a powerful tool to estimate varied cell cycle time and the frequency of different modes of division.

Comments for the author

The manuscript is clearly written and the presented numerical results support conclusions. My main concern, is that the current manuscript lacks enough information to reproduce results of the BP method. Further, the method itself could be better described to facilitate its use by other groups. The model predictions could be possibly tested experimentally (see comments). Taken together, the manuscript with the following comments addressed should be suitable for publication in Development.

Major issues:

1. The BP method was proposed and described in Miguez et al. 2015. In the current manuscript the method is just very briefly described in the Supplementary part. There is very limited information about the input data used in the method (line 320). What were the average parameter values used? How does the error in the input values affect the predicted difference between the rates of progenitor-progenitor and differentiated-differentiated divisions (pp-dd) and the cell cycle length. In practice, what are the confidence intervals in Fig. 3A and 4C. Is the BP method accurate enough to discriminate between the predicted trajectories of pp-dd and cell cycle time for SC and SC+FGF scenarios?

2. The BP along with three alternative methods is tested against a numerical simulation of growing cell population. However, the method limitations are not discussed. Further, I could not find any

© 2020. Published by The Company of Biologists under the terms of the Creative Commons Attribution License (https://creativecommons.org/licenses/by/4.0/).

suggestions about how many sample points should be used and whether they should be equally distributed in time. It would be very helpful to have suggestions concerning planning the experiments (how many time points in a specific time window) to most effectively use the BP method. Adding this information might help other groups in deciding about the applicability of the BP method in the specific experimental system.

3. I miss the experimental validation of the BP method predictions summarized in Fig. 6. If I understand correctly some of the results reported as predictions, e.g. SC vs SC+FGF2 in Fig. 6, were used as input data for the model. Other predictions in Fig. 6 are compared with experimental observations in the literature. I would appreciate explicit notion of what was used as input data, what was related to the already published experimental data, and which observations remain to be tested experimentally as model prediction.

Minor issues:

4. I would mention Branching Process method already in the abstract and highlight its potential significance.

5. The three time points were used, but is it possible to make reasonable predictions with fewer time points as an input? How important is to have equally distributed time points (here every 12h). Is the BP method better performing with more time points but with fewer experiments per time point, or with only a few times with several replicas per point?

6. In line 202 the effect of growth fraction is reported insignificant in line 462 the difference is reported significant with a promise to discuss this discrepancy in the next section. However, I miss a simple one line explanation for this discrepancy. Alternatively, if the reason is complex write it explicitly.

7. Captions for Fig. 6C-D are missing.

8. I don’t see how the effect of growth fraction (line 740) combined with the cell cycle provides increase by 25%. In Fig. 6E the increase of SC+cell cycle and SC+cell cycle+ growth fraction are almost the same (62% vs 65%).

9. Are cell cycle, growth fraction and mode of division independent or interconnected effects within the branching formalism of the BP method? In practice, whether if one of these effects is changed the other two follow or are independent?

10. Line 1028. “the value of each parameter is obtained from gamma distribution”, does it mean all parameters listed in the preceding sentence are drawn from this distribution or only the cell cycle times T_i as suggested by Supp Fig. 2?

11. Line 1029. The spread of the distribution considered is from 0 to 50%. Does 0% case correspond to fully deterministic process with all cell cycles equal and synchronized? What does “similar results” mean in that case?

12. I would appreciate elaborating a bit more about Eq. (1) and (2). Particularly, how one should use these equations with a series of measurements of P and D (n experiments) for different time points (t_0, t_1, …, t_m).

13. Line 1078. Fix 1 <= pp-dd < 0 and 0<= pp-dd <= -1, so the lowest number is on the left, and the largest on the right.

14. Define Ψ. I guess from Miguez 2015, that it is the number of cells undergoing apoptosis, but that should be explained in the text.

15. Consider moving description of the BP method to the Materials and Method section, and expanding the description to make it accessible by non-theoretical community.

© 2020. Published by The Company of Biologists under the terms of the Creative Commons Attribution License (https://creativecommons.org/licenses/by/4.0/).

Reviewer 3

Advance summary and potential significance to field

Ledesma-Terron and colleagues explore the effects of FGF2 signalling on neural progenitors from the developing mouse cortex. Their goal was to extract quantitive data obtained using reductionist cell culture models, and analyse these with new modelling and computational tools to learn new insights into the role of FGF2.

FGF2 is a well-known mitogen for a range of different neural stem and progenitor cells. This study therefore covers similar ground as much older literature, in which fate mapping and growth factor responses of cortical progenitors are explored. The influence of FGF on rate of division is not surprising. Their findings based on the mode of division are perhaps more novel. They also suggest a third role in driving quiescent cells into cycle - which they refer to as the growth fraction.

Overall the data are nicely presented and the quantitative data obtained are of high quality. The innovation and broader interest comes from the modelling strategies and data analysis tools that they have used (branching formalism) – and they raise some interesting points about past EdU analysis and caveats when the cell cycle changes are highly dynamic.

Comments for the author

Mathematical modelling is not my area of expertise, but I agree with them that this is important and interesting. I therefore defer to the other reviewers with expertise in this area to comment on quality and novelty of these methodologies.

I will focus more on the biological relevance. I have several concerns about their interpretation and oversimplification of their conclusions. The conclusions regarding a shift from asymmetric to symmetric modes of division would benefit from independent assays and approaches. The experimental data are rather ’shallow’ and not using state of the art approaches. Also, it would have been straightforward to perform similar experiments with different doses, time-windows combining with EGF etc (see below) and thereby gain greater insights into FGFs impact on radial glia.

My biggest concern is that they have assumed that the only effects of FGF will be on either cell cycle status and rates, or differentiation. However, FGF2 is a well known ventralising signal during early development. Therefore, an additional impact of FGF-2 will be altering the positional identity by cell culture-induced expression of ventral transcription factors. e.g. it is well known that Olig2 and Ascl1 are activated in culture by excessive FGF. Related to this, it is known that FGF induces EGFR expression in these cultures (see paper from Austin Smith lab; Pollard et al., (2008) Mol. Cell. Neurosci. 38, 393–403).

Therefore, the physiological relevance of their findings may not be as simple as just FGF2 alters cortical progenitor proliferation rates and modes of division. I would suggest an alternative: FGF2 induces a ventral radial glial identity, and this might include resetting the cells to a phenotype that is similar to the symmetrically expanding neural progenitors in the ganglionic eminence that have been reported by Magdalena Gotz lab – Pilz G-A, et al (2013) Nat. Commun. 4, 2125. Not inconsistent with their findings - but a different physiological interpretation.

This potential caveat and explanation should at least be discussed. If they were to stain cultures for Olig2 after 24hr in culture they would note this has been artificially activated because of FGF dose. Related to this, they do not explore at all the impact of EGF signalling. In the Introduction they cite a paper by Conti et al in context that suggested 'FGF2 as a the key mitogen'. However, it is EGF that is essential for rapid symmetrical expansion. It is very likely combining FGF with EGF or using EGF alone would give different results to those obtained (in fact EGF is more widely used to expand neural stem/progenitors).

My criticisms therefore relates mainly to the interpretation of their analysis, rather than the quality of the data collected and the modelling. Because this manuscript is being considered at

© 2020. Published by The Company of Biologists under the terms of the Creative Commons Attribution License (https://creativecommons.org/licenses/by/4.0/).

Development, I think the developmental relevance and impact of culture on regional identity is very important and they should address or comment on this in some way.

In summary, this is an interesting methods and modelling study. However, the biological insights are rather limited in novelty, and the physiological relevance potentially a bit unclear. A deeper series of experiments characterising the cellular responses using independent assays, more extensive markers, lineage tracing and expression changes would add a lot and when combined with their modelling and data analysis would be a much stronger study.

First revision

Author response to reviewers' comments

Reviewer 1 Advance Summary and Potential Significance to Field:
This paper presents a thoughtful analysis of the effects of FGF signalling on neural progenitor (radial glia) cell behaviour in vitro. The authors take a fresh approach (using their previously published Branched process tool) to measure and model changes that take place in conditions of increased FGF exposure. In particular, they demonstrate variability associated with calculations of growth fraction and cell cycle length when cumulative labelling approaches are used to determine these values and show greater reproducibility when pulse chase or the branched process approach is applied. This is a useful new analysis of a well-studied phenomenon, some comments for improving the paper are provided below.

Reviewer 1 Comments for the Author:
1) It would be useful to mention the differences found between data generated by distinct cell labelling methods and subsequent analyses in the Abstract. This is a strength of the paper which should be emphasised.

We have updated the abstract in the present version of the text to include the results derived from the analysis using the different cell labelling methods.

EDITED: added a final sentence to the abstract. Abstract has been rewritten to accommodate the 180 word limit after this addition.

2) Key findings presented in the paper are that increased exposure to FGF alters cell division mode (more PP divisions) and that the cell cycle is shortened. This is nicely demonstrated using data on numbers of cells in distinct states identified by immunocytochemistry. Earlier work showing that RG cells accelerate the cell cycle in response to FGF has been published by the Dehay group and should be acknowledged and discussed Lukaszewicz, A. et al 2002 J. Neurosci. 22, 6610-6622.

We sincerely apologize for missing this key reference. We have now added a sentence and this reference in the "Results" when explaining the change in the cell cycle.

EDITED: Added the following sentence to the RESULTS "This reduction in the cell cycle length is in agreement with previous studies by the Dehay group that show that RG cells accelerate the cell cycle in response to FGF". A reference to the work has been also added.

3) As the authors note, another way in which such changes can be studied is to monitor cell behaviour in real time and some relevant work on this has been missed too (Wilcock A. et al 2007 Development 134, 1943-1954). In particular this paper shows that the response of neuroepithelium to FGF exposure is heterogenous: not only is the cell cycle time reduced and more PP divisions observed, but there is an increase in non-apical divisions (which have even shorter cell cycle times) and a breakdown of interkinetic nuclear migration in a subset of cells. The possibility of heterogeneity in response to FGF should be discussed by the authors here – this may relate to differences in the action of FGF in different phases of the cell cycle, as reported by

© 2020. Published by The Company of Biologists under the terms of the Creative Commons Attribution License (https://creativecommons.org/licenses/by/4.0/).

Hitomi, M. and Stacey, D. W. (1999) Curr. Biol. 9, 1075-1084.

Again, we thank the referee for this reference to the Proff. Storey contribution, that has been now added to the text. The referee also raises a very interesting discussion about the possible effect of heterogeneity in the response to FGF. In this paper, they quantify in the chick spinal cord a statistically significant increase in incomplete interkinetic nuclear migration and non-apical divisions, after FGF treatment. They also shown that these basal divisions have a very short cell-cycle (shorter than PP apical). In this direction, to study if the changes in mode and rate of division are due in part to a subset of cells in the population remains a very interesting question.

As we discuss in the paper, both the single cell time-lapse approach (such as the one used by Proff Storey in the chick spinal cord) and our fixed samples approach have pros and cons. For instance, to account for the inherent cell-to-cell variability due to the intrinsic and extrinsic stochasticity in gene expression, a large number of cells have to be tracked simultaneously for a long time. Our fixed-sample immunostaining approach combined with automated image processing allows us to quantify tens of thousands of cells for each condition, providing a good average and hopefully reducing the potential artefacts that inherent cell variability can introduce. The down side is that we may be missing potential heretogeneous effects that may be relevant (such as the one highlighted in Storey's paper). So, our data does not provides information about the potential heterogeneous effect of FGF in RG, maybe because the effect of FGF is different in the different phases of the cell cycle, as the referee points out. We have introduced a detailed discussion in the text, inspired by the referee suggestion. In this direction, and also inspired by this referee, we have performed an analysis of potential heterogeneous effects in the cell cycle length due to different cell rearrangements, as explained in detail in the next point.

EDITED: A discussion about the potential hererogeneous effects of FGF has been added to the discussion. The two references suggested by the referee have also been added to the text.

4) A survey of apico-basal polarity markers would be further informative of the effects of RG exposure to increased FGF in vitro. Antibodies for these are readily available, for example those for N-Cadherin, aPKC, work well. This might reveal whether some or all cells in vitro are behaving in the same way. For example, images for Sox2 in Figure 2A cells in the SC condition are forming a small rosette, which is likely to be largely polarised cells, but the more extensive cell group in the SC+ FGF condition may include cells outwith rosettes lacking A/B polarity, which may equate to the faster dividing cells? Given current constraints on laboratory working, would it be possible to model heterogeneity using an estimated size of a potential A/B polarity lacking cell population in the increased FGF condition? Could the reduced cell cycle time be ascribed to emergence of this cell population over time?

In this comment, the referee proposes a very interesting experiment to test if there is heterogeneity in the A/B polarity. In fact, in both SC and SC+ FGF conditions, some progenitors organize rosette structures (we called it clusters) that accumulate more and more cells as the experiment proceeds. Rather that modelling two populations (we have done it and the branching process gets the average cell cycle of the two populations), we decided to monitor the impact of this heterogeneity by quantifying the change in EdU in the Pulse-Chase experiment (fig 3B) separately for cluster cells and non cluster cells, and compare where in the culture we observe a higher increase in Edu stained progenitors in one versus the other. This way, if EdU staining increases more in the clusters, it would suggest that there is more proliferation in the cluster than outside the cluster. On the other hand, if the increase occurs more outside the clusters (where A/B polarity is presumably lost), then there is faster proliferation outside the cluster. Results are shown in a new supplementary figure 5, where we show representative snapshots of how this division (cluster versus no-cluster) is performed. Bars in the figure show the percentage of EdU+ cells in both time points (pulse and chase), both configurations (cluster and non-cluster) and in both conditions (Sc and SC+FGF). For SC conditions, we can see that the increase in EdU+ cells is very similar between pulse and chase in both clusters and non-clusters.
For SD+FGF, it appears that there is higher increase in aggregates versus non- aggregates, suggesting that maybe cell in aggregates are cycling slightly faster. A more careful analysis taking into account that you start in the "pulse" with around 40% more EdU+ cells initially in clusters,

© 2020. Published by The Company of Biologists under the terms of the Creative Commons Attribution License (https://creativecommons.org/licenses/by/4.0/).

while for the "chase" time point, the difference is slightly reduced (32%) (assuming a simple exponential expansion, the same difference between the "chase" points (40%) should be maintained if both populations cycle at the same speed). In conclusion, these analysis suggests that cells in both conditions are cycling at around the same speed, but a more careful analysis should be performed to check if these small differences observed are representative and statistically significant. We thank the referee again for pointing us in this direction, that we plan to follow in our next paper.

EDITED: Added a new paragraph in the discussion about heterogeneity. Added new supp fig 5.

5) Given the effect of FGF on cell cycle time some more consideration/discussion of the longer cell cycle that is characteristic of asymmetrically dividing neural progenitors should be provided. The known ability of FGF to repress retinoid signalling which is required for expression of cell cycle genes including cdk inhibitors and for expression of neurogenic genes and ultimately neuronal differentiation should also be discussed when considering how FGF influences proliferation.

We agree with the referee that, in fact, two of the consequences of FGF stimulation (shortening the cell cycle and inducing pp divisions) may correspond to two aspects of the same role of FGF. One potential explanations is that, since FGF induces progenitor- progenitor divisions at the expenses of asymmetric divisions, and it has been shown that progenitors undergoing neurogenic divisions have a longer cell cycle than those undergoing proliferative divisions (Calegari et al: J Neurosci 2005, 25:6533-6538.).

On the other hand, there is also evidence that lengthening the neuroepithelial cell cycle can alone be sufficient to trigger differentiation. Based in this hypothesis known as "cell cycle length hypothesis" (from Calegary & Huttner, J Cell Sci 2003, 116:4947-4955), the previously reported effect of FGF in shortening the cell cycle (Lukaszewicz, A. et al 2002 J. Neurosci. 22, 6610-6622) may result in the increase of proliferative divisions. The link between cell cycle length and mode of division is a very interesting topic, and we have previously used this Branching process formalism to study the interplay between mode and rate of division (Míguez, Sci Rep, 5, 13265 (2015), see supplement fig 2). In fact we are currently working in establishing a "physical" link between differentiation and cell cycle by applying forces to constrain growth of a given organ and see the effect in the differentiation dynamics, but is this is very much an ongoing work.

Following the suggestion of the referee, we have added a new paragraph in the discussion focusing on the potential links between the two effects observed after FGF stimulation in shortening cell cycle and promotion pp divisions.

We have also added a paragraph in the discussion to talk about the mutual inhibitor link between FGF and retinoid signalling (nicely reviews by Diez del Corral & Storey (BioEssays 26.8 857, 2004). Since retinoid signalling promotes the expression of inhibitors of CDKs and neurogenic genes, its repression by increased levels of FGF can explain the triple effect of FGF in the mode, rate of divisions and also in the growth fraction.

EDITED: added two paragraphs in the discussion, based on the two very useful suggestions of the referee.

Minor comments

1) Would be good to include the Tbr2 data, which is currently not shown. Tbr2 expressing cells are transit amplifying progenitors which undergo symmetric divisions and ultimately undergo division to generate two neurons. It is important to distinguish between these and NE cells that only divide to produce proliferative progeny.

The immunostaining showing the absence of the Tbr2 positive progenitors in our population is now part of the Supplementary Material.
EDITED: added a new Supp Fig 1 with Tbr2 Immunostaining

© 2020. Published by The Company of Biologists under the terms of the Creative Commons Attribution License (https://creativecommons.org/licenses/by/4.0/).

2) Reconsider colours in 4B (blues too similar) and 6A-D, avoiding red/green.

Colors have been edited following referee suggestions.

EDITED: blue colorbars changed in all figures. Fig 6 edited to facilitate interpretation for colorblind readers.

Reviewer 2 Advance Summary and Potential Significance to Field:
The manuscript “FGF2 modulates simultaneously the mode, the rate of division and the growth fraction in cultures of Radial Glia” investigates how FGF2 affects the differentiation dynamics of Radial Glial progenitors in the in vitro self-renewing culture. By combining experimental and numerical methods the FGF2 is identified to shorten the average cell cycle, promote progenitor-progenitor divisions and increase the growth fraction in the differentiation dynamics of Radial Glia in vitro. The population model of proliferating and differentiating cells is used to compare accuracy of four methods for estimating the cell cycle length. The Branching Process (BP) method is found to provide the most accurate cell cycle estimate among the four considered methods.

The proposed BP method is based on a stochastic formalisms that allows to make predictions about temporal changes in parameters describing growing population of cells. The BP method itself is not restricted to in vitro systems and can be potentially applied to many other in vivo developmental systems. Thus the BP method might be of high interest for stem cell and developmental biology communities, as a powerful tool to estimate varied cell cycle time and the frequency of different modes of division.

Reviewer 2 Comments for the Author:
The manuscript is clearly written and the presented numerical results support conclusions. My main concern, is that the current manuscript lacks enough information to reproduce results of the BP method. Further, the method itself could be better described to facilitate its use by other groups. The model predictions could be possibly tested experimentally (see comments). Taken together, the manuscript with the following comments addressed should be suitable for publication in Development.

Major issues:

1. The BP method was proposed and described in Miguez et al. 2015. In the current manuscript the method is just very briefly described in the Supplementary part. There is very limited information about the input data used in the method (line 320). What were the average parameter values used? How does the error in the input values affect the predicted difference between the rates of progenitor-progenitor and differentiated- differentiated divisions (pp-dd) and the cell cycle length. In practice, what are the confidence intervals in Fig. 3A and 4C. Is the BP method accurate enough to discriminate between the predicted trajectories of pp-dd and cell cycle time for SC and SC+FGF scenarios?

We apologise for provided a reduced amount of information about the BP method and its posibilities. We did so because we were trying to avoid duplicating information from our previous paper, but in doing so, we were probably too brief and some things remained unclear. This new version of the manuscript has a more detailed explanation of the tool, where we put special attention in the questions suggested by the referee. The parameters used as input for the BP are the fitted number of progenitor and differentiated cells from the 11 time points (Fig 2BC, green and red curves) where we quantified the number of progenitor and differentiated cells. In addition, the equations require the average rate of apoptosis (Supp fig 1B) and quiescence (Fig 4B). We have included the 50% confidence intervals in Fig. 3A and 4C, to show that indeed, the BP method can clearly discriminate between the predicted trajectories of pp-dd and cell cycle time for SC and SC+FGF. These confidence intervals have been calculated by fitting the half maximum value of the errors por P and D in Figs 2BC to sigmoidal curves. These values are then fitted to the BP equations to obtain how the value of T and pp-dd deviates assuming an error that is half of the maximum value. The area between the two values of T and pp-dd from the curves that correspond to above and below the half maximal error are used to mark the 50% confidence intervals plotted

© 2020. Published by The Company of Biologists under the terms of the Creative Commons Attribution License (https://creativecommons.org/licenses/by/4.0/).

in the new version of Fig. 3A and 4C. We can see that, although errors are quite large, the intervals for the conditions of SC and SC+FGF do not overlap much. This suggests that the BP method is accurate enough to discriminate between the predicted trajectories of pp-dd and cell cycle time for SC and SC+FGF scenarios.

EDITED: updated the Methods section where we explain the BP tool with more detail. Updated Fig. 3A and 4C with 50% confidence intervals. Explanation of how theses are calculated has been added to the Methods section “Statistics and Data Analysis”. Updated figure caption.

2. The BP along with three alternative methods is tested against a numerical simulation of growing cell population. However, the method limitations are not discussed. Further, I could not find any suggestions about how many sample points should be used and whether they should be equally distributed in time. It would be very helpful to have suggestions concerning planning the experiments (how many time points in a specific time window) to most effectively use the BP method. Adding this information might help other groups in deciding about the applicability of the BP method in the specific experimental system.

Again, we apologize for the lack of details in our explanation of the limitations of the BP method, (we did not want to repeat information that was already published). The discussion has been updated to explain the limitations of the tool (such as the need of absolute values for the number of cells, and the requirement that differentiated cells have to be terminally differentiated).

In terms of the suggestion about the design fo the experiment: the BP method is able to give you the average mode of division and the average values with just two time points. We use 11 data points because we were interested in the time evolution of pp-dd and T. Also, the points do not need to be equispaced in time (in fact, the 11 time points used are not equispaced). The optimal number of time points depends mainly on how fast the number of cells duplicate in your system. As a rule of thumb, we suggest a minimum of five time points for each time the number of total cells duplicates. So, if the number of cells in your system duplicates every 20 hours (as in our conditions of SC+FGF, Fig 2C), you need at least 5 points to obtain a reliable time evolution (we are doing around 7 in this example). This information has been added to the methods section, where we introduce now the Branching Process (it was in supplementary before).

EDITED: updated discussion with the limitations of the PB method. Updated Method sections where we explain the BP tool with details of the experimental design.

3. I miss the experimental validation of the BP method predictions summarized in Fig. 6. If I understand correctly some of the results reported as predictions, e.g. SC vs SC+FGF2 in Fig. 6, were used as input data for the model. Other predictions in Fig. 6 are compared with experimental observations in the literature. I would appreciate explicit notion of what was used as input data, what was related to the already published experimental data, and which observations remain to be tested experimentally as model prediction.

We sincerely apologise for the confusion. There predictions for T (Fig 4C) and pp-dd (Fig 3A) are validated using the numerical model. These predictions are used as input of numerical simulations, and the number of cells resulting from the simulations (thin lines in Fig 6 AB) are compared to the experimental data (reproduced again Fig 6 AB, thick lines). We have updated the text in an effort to explain better the validation process. We have also updated the label of the figure to clarify the figure. We hope that the crucial point of the paper is now more clear in the new version of the manuscript.

EDITED. Label of Fig 6. Explanation of the results shown in Fig 6.

Minor issues:

4. I would mention Branching Process method already in the abstract and highlight its potential significance.

© 2020. Published by The Company of Biologists under the terms of the Creative Commons Attribution License (https://creativecommons.org/licenses/by/4.0/).

A mention of the results derived for the Branching Process has been added to the abstract. EDITED:

Abstract.

5. The three time points were used, but is it possible to make reasonable predictions with fewer time points as an input? How important is to have equally distributed time points (here every 12h). Is the BP method better performing with more time points but with fewer experiments per time point, or with only a few times with several replicas per point?

We apologise for not including this information in the first version of the text, that is now included in the Methods section. As explained in the response to one of the major points of the referee, the BP method is designed to give you the average mode of division (pp-dd) and the average cell cycle length between any two data points. We could use the three points of figure 1 (we will obtain just two values of pp-dd and T, so not enough information of how the values change in time), but we decided to perform more experiments (the 11 points of Fig 2b and C) as input to obtain the temporal evolution of pp-dd and T along the experiment. Also, these points do not need to be equispaced in time (the 11 time points used are not equispaced). In terms of the decision between more replicas or more time points, it depends if your interest is the time evolution (so, more data points) or more reliable values (so, more replicas). This information is now added to the manuscript.

EDITED: added a paragraph in the section corresponding to the explanation of the branching process.

6. In line 202 the effect of growth fraction is reported insignificant in line 462 the difference is reported significant with a promise to discuss this discrepancy in the next section. However, I miss a simple one line explanation for this discrepancy. Alternatively, if the reason is complex write it explicitly.

We apologise for this typo. The reason is nos included in the present version of the manuscript. Basically, our simulations of the cumulative curve processes (such as the ones shown in Fig 5) show that the growth fraction measured with Edu accumulation works well when the cell cycle and mode or division are constant, but it is not accurate when the parameters are variable. This information has been now added to the text.

EDITED: added a paragraph in section "The branching process tool outperforms cumulative curve methods to monitor cell cycle dynamics" to explain the potential source for discrepancy between the Edu data and Ki67 data.

7. Captions for Fig. 6C-D are missing.

We apologise for this mistake, due to the several versions of the manuscript. The caption has been added in the new version of the text.

EDITED: Caption added to fig 6

8. I don't see how the effect of growth fraction (line 740) combined with the cell cycle provides increase by 25%. In Fig. 6E the increase of SC+cell cycle and SC+cell cycle+ growth fraction are almost the same (62% vs 65%).

We apologise for this typo. We have edited the text to clarify this.

EDITED: the text has been edited to correct this typo and to simplify this explanation.

9. Are cell cycle, growth fraction and mode of division independent or interconnected effects within the branching formalism of the BP method? In practice, whether if one of these effects is changed the other two follow or are independent?

© 2020. Published by The Company of Biologists under the terms of the Creative Commons Attribution License (https://creativecommons.org/licenses/by/4.0/).

In the model the three variables are independent, in terms that one can change wile the other remain constant. This is explained in detail in the original paper, where the BP method is derived. In this derivation, the three variables are defined as independent. We have included a sentence in the manuscript to include this information. Of course, in the experiments, they may not be independent, and changes in one of the parameters (cell cycle, form instance) can induce changes in the other. There is a new paragraph in the discussion that talks about potential interplay between mode and rate of division.

EDITED: Added this information in the manuscript. Added paragraph in discussion.

10. Line 1028. “the value of each parameter is obtained from gamma distribution”, does it mean all parameters listed in the preceding sentence are drawn from this distribution or only the cell cycle times T_i as suggested by Supp Fig. 2?

New apologise for this confusion. The means that, for each cell in the simulation, the cell cycle length (T), the probability of differentiation (pp-dd), the probability to enter quiescence (gamma), and the probability to enter apoptosis are obtained from gamma distributed values with an average value set by the input parameters (T, pp-dd, Gamma, and ø). This information is clarified in the text. The description of the model has been moved to the methods section to reach the word limit of the journal.

EDITED: added this information to the Methods section of the manuscript.

11. Line 1029. The spread of the distribution considered is from 0 to 50%. Does 0% case correspond to fully deterministic process with all cell cycles equal and synchronized? What does “similar results” mean in that case?

Thanks for this question. Initially, Progenitor cells in the simulation are always unsyncronized in their cell cycle (randomly distributed). In this case, when 0% distribution of the values of cell cycle, you obtain some small “jumps” in the number of cells occur (when sister cells divide simultaneosly). In this case, the prediction of pp-dd and T has also bumps but in average it works. Since this is not exactly equivalent, we have rewritten the text to remove the 0% standard deviation.

EDITED: Removed the 0% value in the manuscript

12. I would appreciate elaborating a bit more about Eq. (1) and (2). Particularly, how one should use these equations with a series of measurements of P and D (n experiments) for different time points (t_0, t_1, …, t_m).

We apologise for the reduced description of how the equations can be used (a more detailed explanation can be found in the original manuscript Míguez DG, Sci Rep). We have updated the explanation of the branching process (now in methods section) to included information of how the tool works with a series of measurements of P and D (n experiments) for different time points (t_0, t_1, …, t_m). In brief, you can simply use directly the mean of the data points as input, (you obtain discrete values of pp-dd and T between the time points) or you can fit the data to a curve (as we do in this manuscript) and obtain a more smooth value of pp-dd and T. This information has been now added to the text.

EDITED: Added explanation of how to use the equations in the methods section.

13. Line 1078. Fix 1 <= pp-dd < 0 and 0<= pp-dd <= -1, so the lowest number is on the left, and the largest on the right.

This has been fixed. Thank you.

EDITED: change in text following the referee suggestion

© 2020. Published by The Company of Biologists under the terms of the Creative Commons Attribution License (https://creativecommons.org/licenses/by/4.0/).

14. Define Ψ. I guess from Miguez 2015, that it is the number of cells undergoing apoptosis, but that should be explained in the text.

Ψ is a function of the value of pp-dd that is required to account that cells are not synchronised in the experiment. In brief, when the analytical expression of T (eq 2) is derived mathematically from the Branching Process equations of P and D (this step is shown in detain in the original paper Míguez DG, Sci Rep), this is done assuming implicitly that cell decisions in terms of cycling rates are synchronous (which is clearly not the case in real life). We have seen using theoretical studies and modelling that this introduces an error only when the value of pp-dd approaches -1 (i.e., when there is a lot more differentiation than proliferation), but the solution is exact for values of pp-dd>0) Since this correction does not apply to the experimental conditions here (pp-dd is always larger than
0.5 at all times and for all conditions (then the value of Ψ=0 is always equal to 1). To try to simplify the use of the equations, and since the correction does not apply here, we decided for the sake of simplicity to directly substitute Ψ=1 in the equations.

EDITED: Updated eq 2. Updated text to remove the explanation of Ψ.

15. Consider moving description of the BP method to the Materials and Method section, and expanding the description to make it accessible by non-theoretical community.

We have followed the suggestion by the referee and moved the BP method description to the methods. We have also updated the text with a more detailed explanation of the methods, the experimental design and the limitations.

EDITED: moved the BP from "supplementary" to "Methods". Updated the text to include more details.

Reviewer 3 Advance Summary and Potential Significance to Field:
Ledesma-Terron and colleagues explore the effects of FGF2 signalling on neural progenitors from the developing mouse cortex. Their goal was to extract quantitive data obtained using reductionist cell culture models, and analyse these with new modelling and computational tools to learn new insights into the role of FGF2.

FGF2 is a well-known mitogen for a range of different neural stem and progenitor cells. This study therefore covers similar ground as much older literature, in which fate mapping and growth factor responses of cortical progenitors are explored. The influence of FGF on rate of division is not surprising. Their findings based on the mode of division are perhaps more novel. They also suggest a third role in driving quiescent cells into cycle - which they refer to as the growth fraction.

Overall the data are nicely presented and the quantitative data obtained are of high quality. The innovation and broader interest comes from the modelling strategies and data analysis tools that they have used (branching formalism) – and they raise some interesting points about past EdU analysis and caveats when the cell cycle changes are highly dynamic.

Reviewer 3 Comments for the Author:

Mathematical modelling is not my area of expertise, but I agree with them that this is important and interesting. I therefore defer to the other reviewers with expertise in this area to comment on quality and novelty of these methodologies.

I will focus more on the biological relevance. I have several concerns about their interpretation and oversimplification of their conclusions. The conclusions regarding a shift from asymmetric to symmetric modes of division would benefit from independent assays and approaches. The experimental data are rather 'shallow' and not using state

© 2020. Published by The Company of Biologists under the terms of the Creative Commons Attribution License (https://creativecommons.org/licenses/by/4.0/).

of the art approaches. Also, it would have been straightforward to perform similar experiments with different doses, time-windows, combining with EGF etc (see below) and thereby gain greater insights into FGFs impact on radial glia.

My biggest concern is that they have assumed that the only effects of FGF will be on either cell cycle status and rates, or differentiation. However, FGF2 is a well known ventralising signal during early development. Therefore, an additional impact of FGF-2 will be altering the positional identity by cell culture-induced expression of ventral transcription factors. e.g. it is well known that Olig2 and Ascl1 are activated in culture by excessive FGF. Related to this, it is known that FGF induces EGFR expression in these cultures (see paper from Austin Smith lab; Pollard et al., (2008) Mol. Cell. Neurosci. 38, 393–403).

Therefore, the physiological relevance of their findings may not be as simple as just FGF2 alters cortical progenitor proliferation rates and modes of division. I would suggest an alternative: FGF2 induces a ventral radial glial identity, and this might include resetting the cells to a phenotype that is similar to the symmetrically expanding neural progenitors in the ganglionic eminence that have been reported by Magdalena Gotz lab – Pilz G-A, et al (2013) Nat. Commun. 4, 2125. Not inconsistent with their findings - but a different physiological interpretation.

This potential caveat and explanation should at least be discussed. If they were to stain cultures for Olig2 after 24hr in culture they would note this has been artificially activated because of FGF dose.

We sincerely thank the referee for this potential explanation of the results, which is based on the ventralising potential of FGF. Based on this, FGF drives expression of proteins and transcription factors (such as Olig2, Mash1 and Ascl1) (also pointed out in Conti et al, Plos Bio, 2005) that corresponds to a more ventral radial glial identity, such as progenitor cells in the developing ganglionic eminence. In this regions, cells dividing basally outnumber the apical progenitors. This sub-apical progenitors are able to divide more than once, and their cell cycle shortens with each generation (unfortunately, we cannot access the lab to test changes in Olig2 staining after FGF stimulations, but the referee is probably correct that Olig2 may be upregulated, as it has been measured previously for other labs).

This suggests an additional explanation: the increase of FGF drives a more ventral identity, where cells have been shown to divide basally and faster (i.e, more pp divisions and shorter cell cycle). As the referee points out, this is fully consistent with our observations and provides an additional more physiological interpretation of our results.

We sincerely thank the referee for this point. We are including a paragraph in the new version of the text to explain this hypothesis.

EDITED: Added a paragraph in the new version of the manuscript, where we introduce the physiological interpretation suggested by the referee, and some references.

Related to this, they do not explore at all the impact of EGF signalling. In the Introduction they cite a paper by Conti et al in context that suggested 'FGF2 as the key mitogen'.
However, it is EGF that is essential for rapid symmetrical expansion. It is very likely combining FGF with EGF, or using EGF alone would give different results to those obtained (in fact EGF is more widely used to expand neural stem/progenitors).

We apologise for not including the information about the key role of EGF in the establishment of the RG culture in the Austin Smith lab paper (Conti et al). This has been corrected in the new version of the text. In fact, our initial approach was to combine EGF and FGF to study the dynamics of the balance in a differentiation culture versus a proliferating culture. But given that FGF alone induces three simultaneous effects in the dynamics of the culture, the interpretation of the specific role of each signal would be obscured by the combined effect fo FGF and EFG, and by the interplay between both signals. Due to this, and the already large number of figures (6 in the text

© 2020. Published by The Company of Biologists under the terms of the Creative Commons Attribution License (https://creativecommons.org/licenses/by/4.0/).

and 5 as supplementary), we decided to explore the role of EFG and the cooperative interplay when combined with FGF in the switch from a non-expanding to expanding culture of RG in a different paper. This follow up manuscript is in progress, and we hope to resume it as soon as the animal facility is up and running.

EDITED: Sentence changed “established with Fibroblast Growth Factor 2 (FGF2) and EGF as the key molecules that facilitate their expansion”. Added discussion about the role of EGF and the interplay with FGF.

My criticisms therefore relates mainly to the interpretation of their analysis, rather than the quality of the data collected and the modelling. Because this manuscript is being considered at Development, I think the developmental relevance and impact of culture on regional identity is very important and they should address or comment on this in some way.

In summary, this is an interesting methods and modelling study. However, the biological insights are rather limited in novelty, and the physiological relevance potentially a bit unclear. A deeper series of experiments characterising the cellular responses using independent assays, more extensive markers, lineage tracing and expression changes would add a lot and when combined with their modelling and data analysis would be a much stronger study.

We fully agree with the referee that the present paper would be improved if our modelling, theory and experiments is combined with a more detailed focus on the cellular characterisation using lineage tracing and time-lapse movies. Again, due to the already Due to this, and the extension of the manuscript as it is (a bit above the 7000 words limit for development), new decided for this manuscript to focus only on the quantification of the culture to obtain the average dynamics of the culture as a system. We sincerely appreciate the advice of the referee to combine this approach with a more detailed cellular characterization of the differentiation, and we plan to use it in our already mentioned follow-up paper of the interplay between FGF and EGF.

Second decision letter

MS ID#: DEVELOP/2020/189712

MS TITLE: FGF2 modulates simultaneously the mode, the rate of division and the growth fraction in cultures of Radial Glia

AUTHORS: Mario Ledesma-Terron, Nuria Peralta-Canadas, and David G Miguez

Apologies for the delay. One of the referees was late returning their report, but I have now received all the referees reports on the above manuscript, and have reached a decision. The referees' comments are appended below, or you can access them online: please go to BenchPress and click on the 'Manuscripts with Decisions' queue in the Author Area.

The overall evaluation is positive and we would like to publish a revised manuscript in Development, provided that the referees' comments can be satisfactorily addressed. As suggested by Referee 1 please move new Figure S5 into the Results section and cite Lukaszewicz et al., 2002 appropriately.Please also attend to Referee 2's comments in your revised manuscript.

Reviewer 1

Advance summary and potential significance to field

This paper presents a thoughtful analysis of the effects of FGF signalling on neural progenitor

© 2020. Published by The Company of Biologists under the terms of the Creative Commons Attribution License (https://creativecommons.org/licenses/by/4.0/).

(radial glia) cell behaviour in vitro. The authors take a fresh approach (using their previously published Branched process tool) to measure and model changes that take place in conditions of increased FGF exposure. In particular, they demonstrate variability associated with calculations of growth fraction and cell cycle length when cumulative labelling approaches are used to determine these values and show greater reproducibility when pulse chase or the branched process approach is applied. This is a useful new analysis of a well-studied phenomenon.

Comments for the author

In this revision, the authors have adequately addressed most of this reviewer's comments and in particular provide new analysis of potential differences between cell behaviour within and outside neuroepithelial cell clusters. It is unfortunate that further experiments characterising the cell populations analysed +/- FGF cannot currently be undertaken to allow firmer conclusions to be drawn.

Minor points:

1) Lines 835-836 -should include (Lukaszewicz et al., 2002)
Our results show that FGF promotes pp divisions and shortens cell cycle, consistent with findings of (Lukaszewicz et al., 2002) and the hypothesis that proliferative divisions have a shorter cell cycle.

2) Presentation of data in new figure S5 should be in the Results section not the Discussion.

<u>Reviewer 2</u>

Advance summary and potential significance to field

Similar to the original manuscript, in the revised manuscript I find the BP method that provides estimates of temporal changes of cell cycle time and the rate of pp and dd divisions to be potentially a very useful addition to the methods used by stem cell and developmental biology community. Using the BP method combined with single-cell resolution experiments might provide new insights in many studies in which estimation of cell cycle time or the rate of divisions are crucial for final conclusions.

Comments for the author

In the revised manuscript authors have addressed all my major concerns. The Branching Process (BP) method is now described in a way that makes it easier to use by broader community. In particular, the input data is clearly defined and there is discussion how to modify the method to accommodate for different experimental data. Further, the confidence intervals for Fig. 3A and 4C were introduced and explained in a separate section. We can see now that the pp-dd for SC vs SC+FGF conditions overlaps only at early times, and for later stages there is no overlap with the indicated 50% confidence level. The validation process is now also explained more clearly. The numerical simulation is informed with the initial number of cells, growth fraction, apoptosis rate (from measurements), and T and pp-dd (from BP method). The resulting number of progenitors and number of differentiated cells are then compared against sigmoidal fits to the experimental data. The close match between experimental and numerical data confirms that the analytical expressions for T and pp-dd from the BP method are reasonable approximations of the simulated process.

The authors addressed also my minor concerns. The changes included moving BP method description to the Methods section, simplifying the equations (Psi was set to 1), small modifications in the text and correcting the indicated typos. However, I would still recommend to authors to do a careful reading of the modified paragraphs as many typos are still present (e.g. errorbars-> error bars line 981, either … of … -> either … or … line 1078, teh -> the line 1169).

© 2020. Published by The Company of Biologists under the terms of the Creative Commons Attribution License (https://creativecommons.org/licenses/by/4.0/).

Reviewer 3

Advance summary and potential significance to field

The authors have done a good job to address the set of changes proposed by all reviewers.
My concerns were not major and I think they have a improved the manuscript with the changes and highlighted some of the key areas and caveats more clearly.

Comments for the author

No further suggestions. I agree that a deep further functional characterisation and lineage tracing etc would be something that will come in subsequent papers.

Second revision

Author response to reviewers' comments

Dear Editor,

Thank you for your excellent work and for the comments of our resubmission from the referees. In the present version of the text, I have included the few changes suggested by the referees (move the explanation of supporting fig 5 to Results; included reference of Lukaszewicz et al., 2002, corrected typos).

Warm regards,

Third decision letter

MS ID#: DEVELOP/2020/189712

MS TITLE: FGF2 modulates simultaneously the mode, the rate of division and the growth fraction in cultures of Radial Glia

AUTHORS: Mario Ledesma-Terron, Nuria Peralta-Canadas, and David G Miguez
ARTICLE TYPE: Research Article

I am happy to tell you that your manuscript has been accepted for publication in Development, pending our standard ethics checks.

© 2020. Published by The Company of Biologists under the terms of the Creative Commons Attribution License (https://creativecommons.org/licenses/by/4.0/).
